# The role of reactive oxygen in the development of Ramularia leaf spot disease in barley seedlings

**DOI:** 10.1093/aob/mcx170

**Published:** 2017-12-22

**Authors:** Graham R D McGrann, James K M Brown

**Affiliations:** John Innes Centre, Norwich Research Park, Norwich, UK

**Keywords:** *Ramularia collo-cygni*, Ramularia leaf spot, *Hordeum vulgare*, hemibiotroph, senescence, antioxidant system, disease susceptibility, non-functional chloroplasts, endophyte, necrotroph

## Abstract

**Background and Aims:**

*Ramularia collo*-*cygni* is an ascomycete fungus that colonizes barley primarily as a benign endophyte, although this interaction can become pathogenic, causing the disease Ramularia leaf spot (RLS). Factors, particularly reactive oxygen species, that resulted in the transition of the fungus from endophyte to necrotrophic parasite and the development of disease symptoms were investigated.

**Methods:**

Disease development in artificially inoculated seedlings of barley varieties varying in partial resistance to RLS was related to exposure to abiotic stress prior to inoculation. Histochemical and molecular analysis determined the effect of *R. collo*-*cygni* colonization on accumulation of reactive oxygen species and antioxidant gene expression. Development of RLS on barley lines defective in antioxidant enzymes and with altered redox status or non-functional chloroplasts was compared with the accumulation of fungal biomass to determine how these factors affect disease symptom expression.

**Key Results:**

Exposure to abiotic stress increased symptom development in all susceptible and most partially resistant barley varieties, in association with greater hydrogen peroxide (H_2_O_2_) levels in leaves. Decreased activity of the antioxidant enzymes superoxide dismutase and catalase in transgenic and mutant plants had no effect on the disease transition, whereas manipulation of H_2_O_2_ levels during asymptomatic growth of the fungus increased disease symptoms in most susceptible varieties but not in partially resistant plants. Barley mutants that undergo rapid loss of green leaf area when infected by *R*. *collo*-*cygni* or albino mutants with non-functional chloroplasts showed reduced development of RLS symptoms.

**Conclusions:**

These results imply that in seedlings the pathogenic transition of the normally endophytic fungus *R*. *collo*-*cygni* does not result from senescence as such, but rather is promoted by factors that result in changes to host reactive oxygen species. Barley varieties vary in the extent to which these factors promote RLS disease.

## INTRODUCTION

Ramularia leaf spot (RLS) is a late season disease that has emerged as a major problem in barley production in Europe, South America and New Zealand over the last 20 years (Havis *et al.*, 2015; McGrann and Havis, 2017). The pathogen, the Dothideomycete fungus *Ramularia collo*-*cygni* (McGrann *et al.*, 2016), is transmitted both in infected seeds and by spore dispersal between plants (Stabentheiner *et al.*, 2009; Havis *et al.*, 2014). *Ramularia collo*-*cygni* grows initially as an endophyte and can remain in this asymptomatic state for several weeks but, after a long period of latent development, it can then undergo a developmental switch to become an aggressive necrotrophic pathogen (Kaczmarek *et al.*, 2017). In diseased plants, RLS symptoms typically develop on leaves towards the end of the growing season after ear emergence, although symptoms can be observed earlier in the season if environmental conditions are especially conducive to the disease (Harvey, 2002; McGrann and Havis, 2017). The risk of RLS epidemics is predicted to increase as the global climate changes (West *et al.*, 2012), but current understanding of what factors trigger the transition of *R*. *collo*-*cygni* from benign endophyte to necrotrophic pathogen is limited.

Host–endophyte interactions can be commensal, mutualistic or antagonistic (Saikkonen *et al.*, 1998). External conditions that can trigger the switch from endophyte to parasite include an imbalance in nutrient exchange, leaf ageing and senescence, or when the host is under stress (Saikkonen *et al.*, 1998; Schulz and Boyle, 2005; Rodriguez and Redman, 2008). As the agent of a late season disease, the shift of *R*. *collo*-*cygni* from endophyte to pathogen has been associated with changes in host development from vegetative to reproductive stages and with a decline in the host antioxidant system during monocarpic senescence (Schützendübel *et al.*, 2008). However, as most RLS-susceptible elite barley varieties tend to senesce early as a result of high levels of disease, it is difficult to ascertain if there is a causal relationship between senescence and RLS (Oxley *et al.*, 2008). Exposure to high light stress increased the severity of RLS symptoms in controlled-environment experiments on a range of spring barley varieties and the model grass *Brachypodium distachyon* (Makepeace *et al.*, 2008; Peraldi *et al.*, 2014), indicating that the fungal switch to the pathogenic phase may be intimately linked to the host response to its surrounding environment.

Interactions between host genotype and the local abiotic environment can also affect the outcome of an endophyte’s association with its host plant (Saikkonen *et al.*, 1998). Transgenic barley plants which overexpressed *Stressed-related NAC1* (*SNAC1*), encoding a transcription factor involved in drought tolerance in cereals, were more resistant to RLS (McGrann *et al.*, 2015*a*). Reduced RLS was associated with delayed leaf senescence in plants overexpressing *SNAC1*, further suggesting a link between the host signalling pathways that regulate the abiotic stress response and the development of this disease. Additional host genetic components have been implicated in the pathogenic transition of *R*. *collo*-*cygni* from endophyte to aggressive necrotroph. The broad-spectrum barley powdery mildew resistance gene *mlo*, which also regulates a spontaneous lesion mimic phenotype (Wolter *et al.*, 1993) and is associated with accelerated leaf senescence and host cell death (Piffanelli *et al.*, 2002), is a major genetic factor conferring susceptibility to RLS (McGrann *et al.*, 2014). Two genes required for *mlo*-mediated mildew resistance, *ROR1* and *ROR2*, also control the expression of RLS symptoms but not the accumulation of fungal biomass, implicating the *MLO* pathway in the pathogenic transition of *R*. *collo*-*cygni*. Mis-regulated host cell death in barley lesion mimic mutants elicits diverse effects on the switch from a symptomless state to disease, and in some, but not all, cases increases growth of *R*. *collo*-*cygni* (McGrann *et al.*, 2015*b*). Furthermore, a gain-of-function mutation of the cereal dwarfing gene *Slender 1*, encoding a DELLA protein, restricts cell death and increases RLS symptoms (Saville *et al.*, 2012), whereas mutants of a second dwarfing gene *Brassinosteroid insensitive 1* show no effect on RLS (Goddard *et al.*, 2014).

Cell death, leaf senescence and exposure to abiotic stress factors increase levels of reactive oxygen species (ROS) such as superoxide and hydrogen peroxide (H_2_O_2_) through breakdown of the chloroplast, photorespiration in peroxisomes and loss of functional integrity of photosystem II. Altered ROS status causes an imbalance to the redox system, resulting in oxidative stress to the plant (Zimmermann and Zentgraf, 2005). Tight regulation of ROS levels through the action of antioxidant enzymes such as catalase, superoxide dismutase and ascorbate peroxidase, and non-enzymic antioxidants such as glutathione and ascorbate maintains redox balance and prevents cellular damage (Foyer and Noctor, 2005). ROS also play an important role in cellular signalling to enable plants to respond rapidly to changes in the external environment (Suzuki *et al.*, 2012; Saxena *et al.*, 2016). Whether or not oxidative stress has a direct effect on *R*. *collo*-*cygni* development is not understood, but ROS levels are affected by the functions of *mlo* (Piffanelli *et al.*, 2002), *ROR* genes (Hückelhoven *et al.*, 2000), DELLA proteins (Achard *et al.*, 2000) and *SNAC1* (You *et al.*, 2013), all of which affect the biology of this pathosystem.

The research reported here investigated how factors that can cause endophytic fungi to become pathogenic, such as abiotic stress, senescence and ROS, influence the development of RLS symptoms in artificially inoculated seedlings. As in most plant diseases, resistance to RLS is quantitative, and breeding has selected varieties with higher partial resistance (Havis *et al.*, 2015). Variation in the pathogenic transition between barley varieties which vary in susceptibility to RLS was investigated. Barley lesion mimic and albino mutants were used to examine how ROS, cell death and functional chloroplasts affect expression of RLS symptoms as *R. collo*-*cygni* makes its transition from a benign endophyte to an aggressive necrotrophic pathogen.

## MATERIALS AND METHODS

### Plant material

Host responses to RLS were investigated in a collection of spring barley varieties differing in their susceptibility to the disease. The variety Power has strong partial resistance to RLS, whilst Braemar is highly susceptible (Oxley *et al.*, 2008, McGrann *et al.*, 2014). Golden Promise, which is somewhat less susceptible than Braemar, was also included in these experiments. Other spring barley varieties used in this study are listed in Supplementary Data Table S1.

RPr 74/9 is a catalase-deficient mutant of the barley variety Maris Mink (Kendall *et al.*, 1983). Growth inhibition and development of spontaneous necrotic lesions on the leaves were observed under standard glasshouse conditions, but were not visible on RPr 79/4 plants when grown under standard growth room light levels of 220 μmol m^–2^ s^–1^ used in the experiments described here. RNA interference (RNAi) line 161–7- 11 has reduced transcript levels of the barley *Copper/Zinc superoxide dismutase 1* (*CSD1*; Lightfoot *et al.*, 2017). The *Bipolaris sorokiniana tolerant 1* (*bst1*) mutants have resistance to the spot blotch pathogen *Cochliobolus sativus* (anamorph *Bipolaris sorokiniana*; Persson *et al.*, 2008, 2009). *albostrians* is a barley albino mutant that is blocked in chloroplast development. This recessive mutation is inherited maternally and produces plants that segregate in their leaf phenotype, with either completely green, completely white or green and white striped leaves, all of which are genetically identical except for plastid differentiation (Hess *et al.*, 1994). White leaves contain only traces of chlorophyll and carotenoids, lack 70S ribosomes and are photosynthetically inactive (Hess *et al.*, 1994).

### Growth conditions and abiotic stress treatments

Plants were grown in Levington F2 compost (Scotts Professional, Ipswich, UK) with a 16/8 h day/night photoperiod at day/night temperatures of 18/12 °C in a controlled-environment room (Sanyo) supplemented with 220 μmol m^–2^ s^–1^ fluorescent lighting. Plants were watered as required to keep the potting medium moist but not saturated. High light stress was applied by growing plants in a growth cabinet (Snijders Scientific, Tilburg, The Netherlands) under the conditions described above except that 700 μmol m^−2^ s^−1^ fluorescent light was supplied for the entire growing period. Plants were waterlogged by placing plant pots into trays filled with 4–5 cm of water from 8 d post-sowing for 6 d until pathogen inoculation.

### Ramularia collo-cygni inoculation

*Ramularia collo-cygni* isolate Rcc09B4 was maintained and inoculum prepared as previously described (Makepeace *et al.*, 2008; Peraldi *et al.*, 2014). Barley seedlings at GS11–12 (Zadoks *et al.*, 1974) were inoculated evenly with a fine mist at a rate of 10 mL of inoculum on 50 plants (Makepeace *et al.*, 2008). Following inoculation, plants were placed under plastic covers to maintain high relative humidity (80–100 %) in the dark for 48 h and then grown under light levels of 220 μmol m^−2^ s^−1^ for the duration of the experiment. Disease development was assessed over the duration of the experiment as previously described, and disease severity was summarized as the area under the disease progress curve (AUDPC) as a percentage of the maximum AUDPC possible (McGrann *et al.*, 2014). RLS progression on Power, Braemar and Golden Promise was scored as the average disease level per pot from five pots in each experiment. Data on the effects of abiotic stress treatments on RLS was scored on ten plants per variety in each treatment. Disease was scored on at least five plants of each of the *bst1* mutants per experiment. As the *albostrians* mutants segregated for leaf phenotype, 40 seeds were sown in to a 20 × 15 × 5 cm tray for inoculation. Across five independent inoculation experiments, 18.7 % of plants had green leaves, 69.7 % had green and white striped leaves and 11.6 % had white leaves. For all pathology experiments, disease data were collected from a minimum of three separate experiments with independent inoculations.

### Light microscopy of *Ramularia collo-cygni* infection

For microscopic observation of *R. collo-cygni* development, inoculated prophyll leaves were placed in 70 % ethanol until the chlorophyll was cleared. Each leaf was cut into two sections (tip and base) and stained with aniline blue [0.1 % (w/v) in lactoglycerol; Sigma]. Fungal development was quantified using bright-field microscopy (Nikon 800 Eclipse; Nikon Precision Europe GmbH, Langen, Germany) under ×40 magnification. In each field of view where *R. collo-cygni* hyphae were visible, all stomata present were scored for the number of (1) stomata that were penetrated by hyphae; (2) stomata from which conidiophore structures were erupting; (3) penetrated stomata which were surrounded by cells that showed reddish-brown coloured necrosis; and (4) non-penetrated stomata. At least two leaves of each genotype at each time point were scored, with a minimum of 300 stomata in total assessed on each leaf. Samples for light microscopy were collected from Braemar, Power and Golden Promise plants in three independent *R*. *collo*-*cygni* inoculation experiments at 5, 10, 15, 18 and 21 days post-inoculation (dpi).

### Histochemical staining of ROS production

To assess the production of ROS during RLS development, five leaves were sampled at 5, 10, 15, 18 and 21 dpi with each of two treatments, either inoculated with Rcc09B4 or mock-inoculated with potato dextrose broth. Leaves were stained with 3’,3-diaminobenzidine (DAB; Sigma) to detect peroxide precipitation (Thordal-Christensen *et al.*, 1997) and with nitroblue tetrazolium (NBT; Sigma; Adam *et al.*, 1989) for superoxide detection. After staining, leaves were cleared in ethanol:lactic acid:glycerol (3:1:1) until transparent and then photographed. The amount of brown or blue staining visible on each leaf following DAB or NBT treatment, respectively, was quantified from the photographs using ImageJ (Abramoff *et al.*, 2004: https://www.fmhs.auckland.ac.nz/assets/fmhs/sms/biru/docs/Colour_Analysis_Tools_in_ImageJ.pdf) and expressed as a proportion of the total leaf area. Samples for both staining methods were collected from three independent *R*. *collo*-*cygni* inoculation experiments.

### Quantitative PCR analysis of *R. collo-cygni* DNA and host transcript levels

*Ramularia collo-cygni in planta* DNA levels were quantified using quantitative PCR (qPCR) (Taylor *et al.*, 2010). Changes in target gene transcript levels following *R*. *collo-cygni* inoculation or in uninoculated plants were assessed by quantitative reverse transcription–PCR (qRT–PCR) using gene-specific primers as previously described (McGrann *et al.*, 2009, 2015*a*; Colebrook *et al.*, 2012; Supplementary data Table S2). Samples for qPCR of fungal DNA levels and host transcriptional changes were collected 5, 10, 15, 18 and 21 dpi from at least three independent *R*. *collo*-*cygni* inoculation experiments. Five leaves per time point were collected for qPCR, whereas two replicates each consisting of two leaves were collected for qRT–PCR analysis at each time point.

### *In vitro* fungal growth assays

Fungal growth inhibition was assessed by measuring culture diameter extending from a 5 mm agar plug of each fungus on PDA (potato dextrose agar) medium supplemented with 1, 5, 10, 20, 50 or 100 mm H_2_O_2_; 100 μm methyl viologen (Sigma); 5 mm CaCl_2_ (Sigma); 50 mm LiCl (Sigma); and 2000 U ml^–1^ catalase (Sigma); or 5 mm of the irreversible catalase inhibitor 3-amino-1,2,4-triazole (3-AT; Sigma). As each fungus grew at a different rate, the effects of the oxidative stress-inducing agents on each pathogen were assessed at the following time points: *R*. *collo-cygni* (isolate Rcc09B4) at 42 d, *Fusarium culmorum* (isolate Fu42) at 5 d, *Magnaporthe oryzae* (isolate Guy11) and *Oculimacula yallundae* (isolate P149) at 21 d and *Botrytis cinerea* (isolate B05:WT) at 3 d. A minimum of three independent experiments were performed for all *in vitro* growth assays, with fungal growth measured on six replicate plates per experiment.

### Leaf infiltration with catalase, H_2_O_2_, 3-AT and water

To test the effect of H_2_O_2_ status on the development of RLS symptoms, prophyll leaves of barley genotypes were infiltrated with 100 μL of catalase (2000 U ml^–1^; Sigma), H_2_O_2_ (5 mm; Sigma), 3-AT (5 mm; Sigma) or water (control) at 5, 7 and 10 dpi with Rcc09B4. Leaves were infiltrated through a puncture wound at the distal end using a needleless syringe as described previously (Colebrook *et al.*, 2012). Each compound was infiltrated into five leaves per variety at each time point. Disease progression was assessed as described above. Data were collected from three independent *R*. *collo*-*cygni* inoculation experiments.

### ROS-induced cell death assays

Plants were grown under standard light levels (220 μmol m^–2^ s^–1^) for 14 d. The sensitivity of different barley genotypes to ROS was assessed using 200 mm alloxan (Sigma), which is an H_2_O_2_ donor, 100 mm menadione (Sigma), a mitochondrial superoxide donor, and 25 μm methyl viologen (Sigma), a chloroplastic superoxide donor, as described previously (McGrann *et al.*, 2015*a*). Treated leaves were incubated in boxes at room temperature under constant light (15–20 μmol m^–2^ s^–1^) for 96 h. After incubation, each box was photographed and the lesion size measured using ImageJ. Lesion size was assessed on at least six leaves from a minimum of three independent experiments.

### Dark-induced senescence assays

Plants were grown under standard light levels or at high light for 14 d. Dark-induced senescence was assessed through measurements of relative chlorophyll content of the prophyll leaves of each genotype using Chlorophyll Meter SPAD-502 (Konica Minolta, Warrington, UK). Measurements were taken from six leaves per variety in a minimum of three independent experiments at 0, 2, 4 and 6 d of dark treatment as described previously (McGrann *et al.*, 2015*a*).

### Data analysis

All data were analysed using GenStat version 14 (Payne *et al.*, 2009). Unless stated otherwise, data were analysed by analysis of variance (ANOVA) using general linear models that accounted for the variation attributable to different factors used in each experiment. Disease data from all *R*. *collo*-*cygni* inoculation experiments were Logit+ transformed (McGrann *et al.*, 2014) prior to analysis. Data from the microscopy and ROS staining experiments were analysed using generalized linear models. Each score category from the microscopy experiments was analysed separately as a proportion of the total number of stomata scored on each leaf using a binomial distribution as the link function. Data on DAB- and NBT-stained samples were log transformed prior to analysis. Dark-induced senescence data were analysed with a linear mixed model of repeated measures using the uniform correlation/split plot in time covariance matrix (McGrann *et al.*, 2015*a*). Fixed factors were day, experiment, genotype and the interactions between them, with the leaf × day interaction term as the random factor. After modelling, treatments were compared using *t*-test probabilities and applying Fisher’s least significant difference to unplanned comparisons.

## RESULTS

### Development of Ramularia leaf spot in resistant and susceptible varieties

**Fig. 1.**
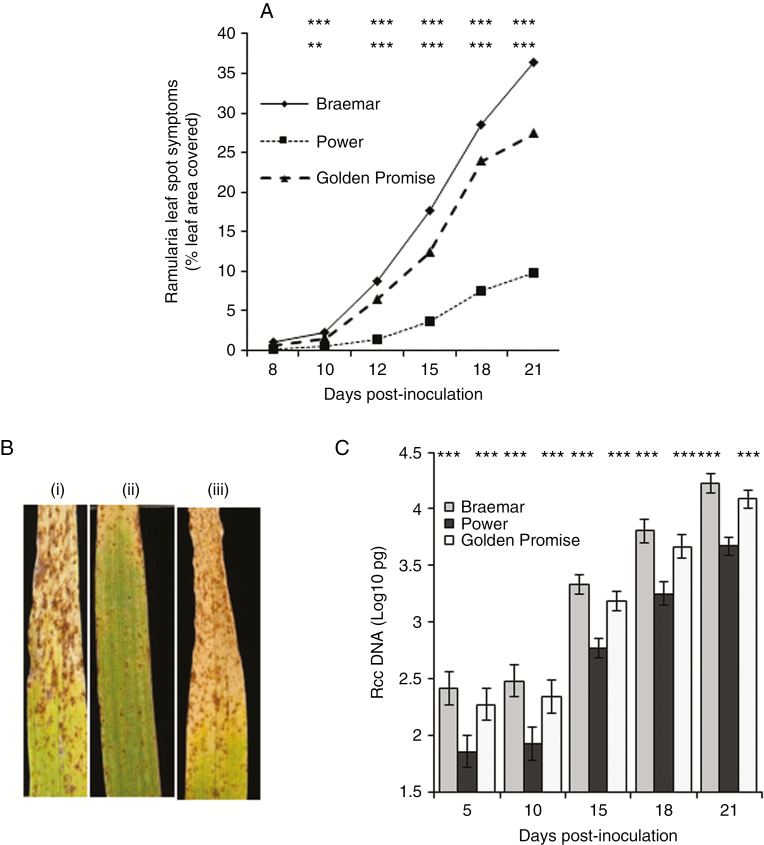
Ramularia leaf spot development on the spring barley varieties Braemar, Power and Golden Promise. (A) Symptom development on prophyll leaves over the infection time course. (B) Typical disease symptoms on Braemar (i), Power (ii) and Golden Promise (iii) 21 days post-inoculation. (C) *Ramularia collo*-*cygni* DNA levels in prophyll leaves over the infection time course measured by qPCR. Error bars indicate ±1 s.e. Data were analysed by general linear modelling. ***P <0.001; **0.001 < *P* < 0.01; *0.01 < *P* < 0.05.

Symptoms of RLS were first visible as small brown pepper spots on the prophyll leaf of inoculated plants approx. 8–10 dpi. Subsequent development of the disease was rapid on the highly susceptible variety Braemar but much slower on Power, which has high partial resistance (Fig. 1A). RLS development on the variety Golden Promise was similar to that on Braemar, although final levels of disease were slightly lower (Fig. 1B). As the disease progressed, the small spots enlarged to form typical RLS lesions that began to coalesce from 15 dpi onwards. The later stages of RLS symptom development were associated with leaf senescence, particularly in susceptible varieties (Fig. 1B). Transcript levels of chlorophyll *a*/*b*-binding protein were reduced approx. 2-fold or more in *R*. *collo*-*cygni*-infected leaves of the susceptible varieties Braemar and Golden Promise, concurrent with the onset of disease symptom development at 10 dpi, but not in Power, which has strong partial resistance to RLS (Supplementary Data Fig. S1). *Ramularia collo*-*cygni* genomic DNA levels significantly increased with time after inoculation in all three varieties tested (*P* < 0.001; Fig. 1C). There was significantly more fungal DNA in the susceptible variety Braemar than in the resistant variety Power (*P* < 0.001).

**Fig. 2.**
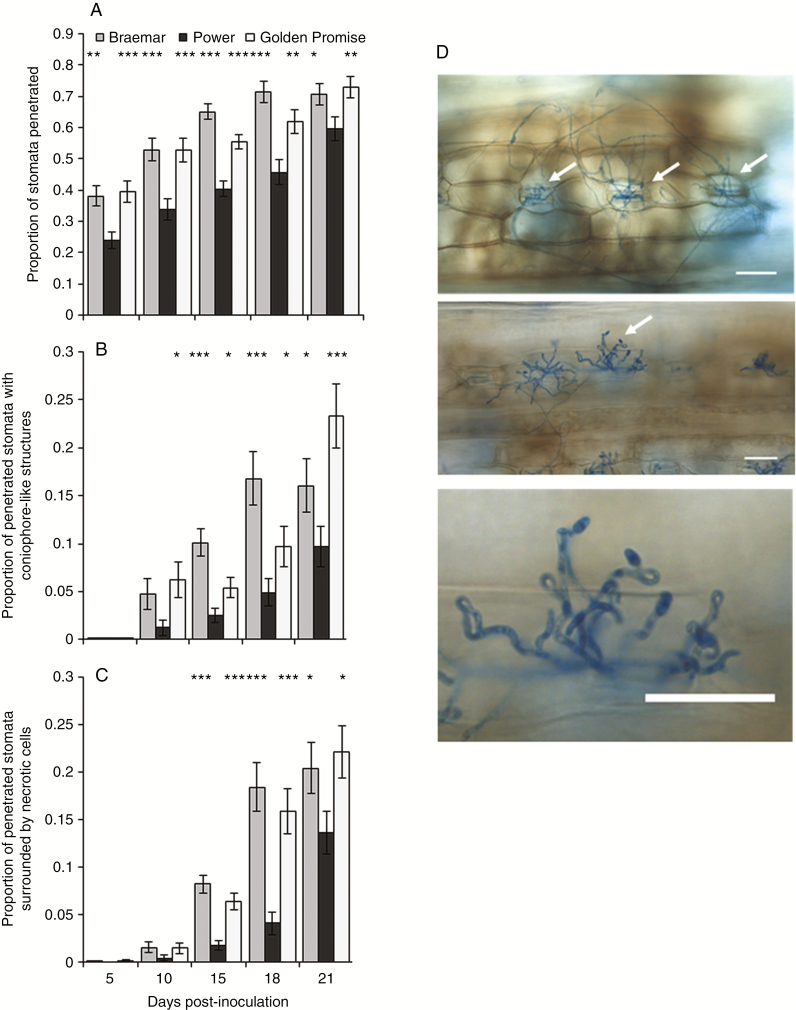
Microscopic comparison of *Ramularia collo*-*cygni* infection in the spring barley varieties Braemar, Power and Golden Promise during an infection time course. (A) Proportion of stomata penetrated by *R. collo*-*cygni* hyphae. (B) Proportion of penetrated stomata with conidiophore-like structures emerging. (C). Proportion of penetrated stomata surrounded by reddish-brown necrotic areas. Error bars indicate ±1 s.e.. (D) Images of microscopic development of *R. collo*-*cygni* infection under ×20 magnification. (i) Stomata penetrated by *R*. *collo*-*cygni* hyphae (arrows) surrounded by reddish-brown necrotic regions. (ii) *Ramularia collo*-*cygni* conidiophores (arrows) erupting from stomata. (iii) Close-up of *R*. *collo*-*cygni* conidiophore highlighting the characteristic swan-neck shape. Scale bar = 50 μm. Data were analysed using a generalized linear model of binomial proportions. ***P < 0.001; **0.001 < *P* < 0.01; *0.01 < *P* < 0.05.

*Ramularia collo-cygni* development on partially resistant and susceptible varieties was visually assessed using light microscopy (Fig. 2A–D). The frequency of stomata penetrated by fungal hyphae increased over time in both varieties but was significantly higher in Braemar throughout the infection time course than in Power (Fig. 2A; *P* < 0.05). Emergence of conidiophore-like structures was rarely observed before 10 dpi but significant differences between resistant and susceptible varieties were recorded from 10 dpi onwards (Fig. 2B; *P* < 0.05). A few regions of reddish-brown cellular necrosis were observed at 5 dpi prior to the appearance of macroscopic disease lesions on the leaf. Larger areas of necrosis were scored as the symptoms developed, with significant differences between Power and Braemar from 15 dpi onwards (Fig. 2C; *P* < 0.05). *Ramularia collo*-*cygni* development in Golden Promise was similar to that observed in Braemar (Fig. 2A–D).

### Abiotic stress and Ramularia leaf spot development

**Fig. 3.**
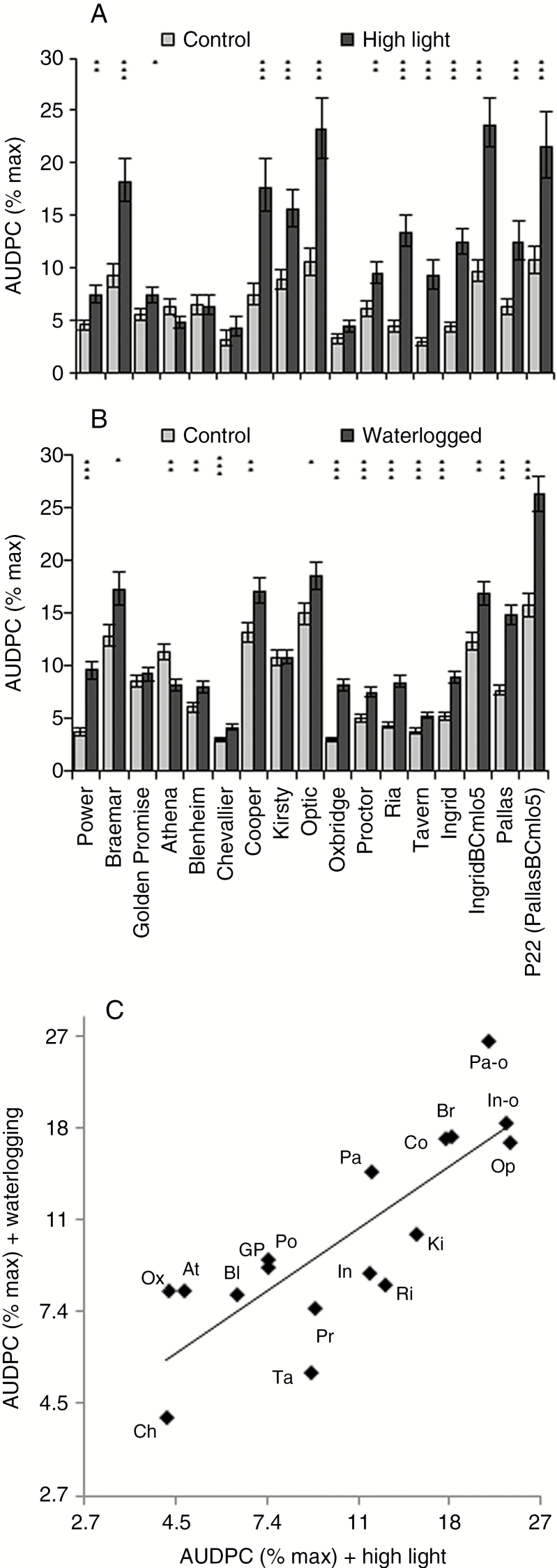
Effect of abiotic stress on Ramularia leaf spot development. Different spring barley varieties were grown under high light conditions (A) or waterlogged for 6 d (B) before inoculation with *Ramularia collo*-*cygni*. Disease development on abiotic stress-treated plants (dark grey bars) was compared with control plants (light grey bars) grown under normal controlled-environment room conditions. Disease is expressed as the area under the disease progress curve (AUDPC). Data were analysed by general linear modelling. ***P < 0.001; **0.001 < *P* < 0.01; *0.01 < *P* < 0.05 for comparisons between stress-treated and control plants. (C) Comparison of varieties’ disease scores under the two abiotic stresses. Codes are the first two letters of the varieties’ names; In-o, Pa-o: *mlo5* near-isogenic lines of Ingrid and Pallas, respectively. Error bars indicate ± 1 s.e.

In a larger panel of barley varieties, exposure to high light levels before inoculation increased disease symptom development (*P* < 0.001) although there was a significant interaction between the stress treatment and variety (Fig. 3A; *P* < 0.001). To assess whether this stress-induced disease increase was specific to high light or a more general response to abiotic stress, a waterlogging treatment was also tested. Similar to the high light treatment, most varieties that had been waterlogged prior to inoculation showed significantly increased levels of RLS (Fig. 3B; *P* < 0.001). Pre-inoculation exposure to abiotic stresses increased disease severity in both RLS-susceptible and partially resistant varieties, with a high correlation between varieties’ susceptibility to RLS under different abiotic stresses [Fig. 3C; correlation (*r*) between mean logit-transformed RLS scores = 0.81, *P* < 0.001]. Varieties’ responses to the two stresses varied; notably, the resistant variety Athena exhibited reduced RLS symptoms following exposure to waterlogging or high light before inoculation (Fig. 3).

To assess if there was a link between high light stress, senescence and varietal susceptibility to RLS dark-induced senescence, a method for studying the physiological mechanisms of leaf senescence in plants (Gan and Amasino, 1997) was used. Leaves from plants grown under high light senesced significantly earlier than control leaves for all the varieties tested (*P* < 0.001; Supplementary Data Fig. S2). We also observed significant differences in dark-induced senescence between genotypes (*P* < 0.001) but no link between the rate of senescence and varietal susceptibility to RLS (Supplementary Data Fig. S2).

### ROS accumulation during Ramularia leaf spot development

**Fig. 4.**
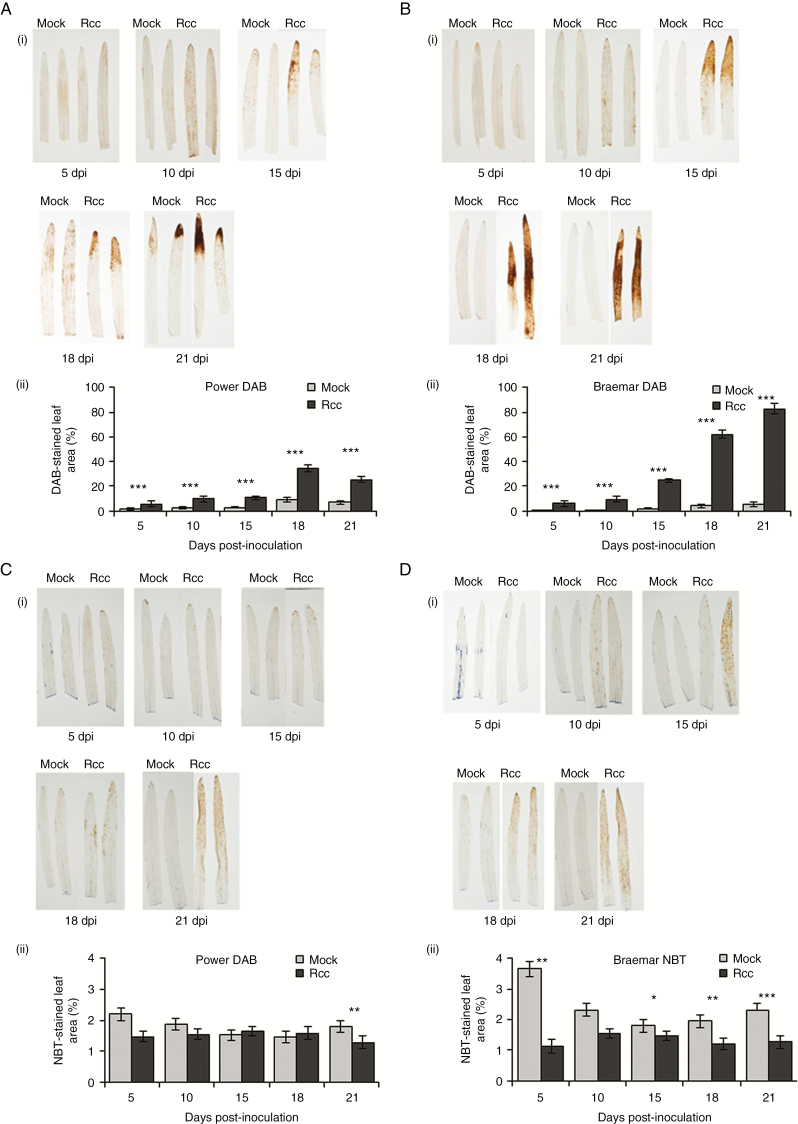
Reactive oxygen species accumulation in Power and Braemar following *Ramularia collo*-*cygni* inoculation. 3',3-Diaminobenzidine (DAB) staining for peroxide accumulation in Power (A) and Braemar (B). Representative images of DAB-stained leaves (i) and proportion of leaves stained with DAB (ii) in mock- and *R*. *collo*-*cygni*-inoculated leaves. Nitroblue tetrazolium (NBT) staining for superoxide accumulation in Power (C) and Braemar (D). Representative images of NBT-stained leaves (i) and proportion of leaves stained with NBT (ii) in mock- and *R*. *collo*-*cygni*-inoculated leaves. Error bars indicate ±1 s.e. Data were analysed using a generalized linear model. ***P < 0.001; **0.001 < *P* < 0.01; *0.01 < *P* < 0.05 for differences between inoculated and mock-inoculated samples.

Staining with DAB and NBT was used to assess the production of H_2_O_2_ and superoxide, respectively, during RLS development in partially resistant and susceptible varieties. Rcc09B4 inoculation increased the proportion of DAB-stained leaf area over time in all genotypes tested (*P* < 0.001). More brown staining following DAB treatment to reveal peroxide was observed on inoculated leaves of the susceptible variety Braemar (Fig. 4B) than on the more resistant variety Power (Fig. 4A) from 15 dpi onwards (*P* < 0.001). Strong DAB staining, comparable with that on Braemar, was also observed on Golden Promise (Supplementary Data Fig. S3). Rcc09B4 inoculation slightly reduced the overall level of NBT staining on Braemar compared with mock-inoculated control leaves (*P* < 0.001; Fig. 4D). On Power, the reduction in NBT staining following inoculation was only statistically significant at 21 dpi (*P* = 0.005; Fig. 4D). Overall there was no significant difference in NBT staining between Power and Braemar (*P* = 0.065), but there was a significant three-way interaction of variety, treatment and time point, implying that the rate of accumulation of NBT differed between the two varieties (*P* <0.001). This was largely because mock-inoculated Braemar stained more heavily with NBT at 5 dpi although the area stained was much smaller in all cases than with DAB. NBT staining was also lowered by Rcc09B4 inoculation in Golden Promise, although this was only significant at 5 dpi (*P* = 0.008; Supplementary Data Fig. S3).

### *Ramularia collo-cygni* tolerance to conditions inducing oxidative stress

**Fig. 5.**
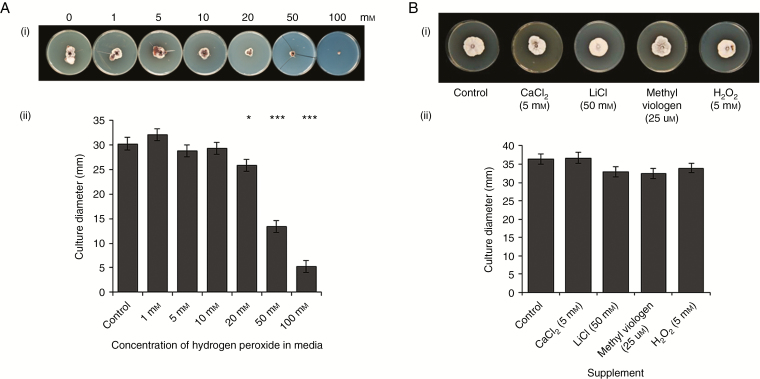
*Ramularia collo*-*cygni in vitro* growth on oxidative stress-inducing media. (A) Growth of *R*. *collo*-*cygni* on potato dextrose agar (PDA) media supplemented with different concentrations of H_2_O_2_. Representative image (A) and measurements of *R*. *collo*-*cygni* culture diameter after 42 d growth. (B) Effect of oxidative stress-inducing media on *in vitro* growth of *R*. *collo*-*cygni*. Representative image (i) and measurements (ii) of *R*. *collo*-*cygni* culture diameter after 42 d growth on PDA media supplemented with CaCl_2_ (5 mm), LiCl (50 mm), H_2_O_2_ (5 mm) and methyl viologen (25 µm). Error bars indicate ±1 s.e. Data were analysed by general linear modelling. ***P < 0.001; **0.001 < *P* < 0.01; *0.01 < *P* < 0.05 for differences from control plates with no supplement.

As elevated H_2_O_2_ levels coincided with the later stages of RLS symptom development, the tolerance of *R*. *collo*-*cygni* to this ROS was tested in an *in vitro* growth assay. Growth of Rcc09B4 was significantly inhibited only at H_2_O_2_ concentrations >10 mm (Fig. 5A). Furthermore, *R*. *collo-cygni in vitro* growth was not significantly inhibited compared with control plates by conditions expected to induce oxidative stress to the fungus including salinity and methyl viologen (Fig. 5B; *P* = 0.099 for comparisons of treatments with controls). In contrast, *in vitro* growth of the fungi *M. oryzae*, *B. cinerea*, *F. culmorum* and *O. yallundae* was inhibited by some or all of the stress-inducing compounds LiCl, CaCl_2_, methyl viologen and H_2_O_2_ (Supplementary Data Fig. S4).

### Response of antioxidant and defence transcripts during *R. collo-cygni* infection of resistant and susceptible varieties

**Fig. 6.**
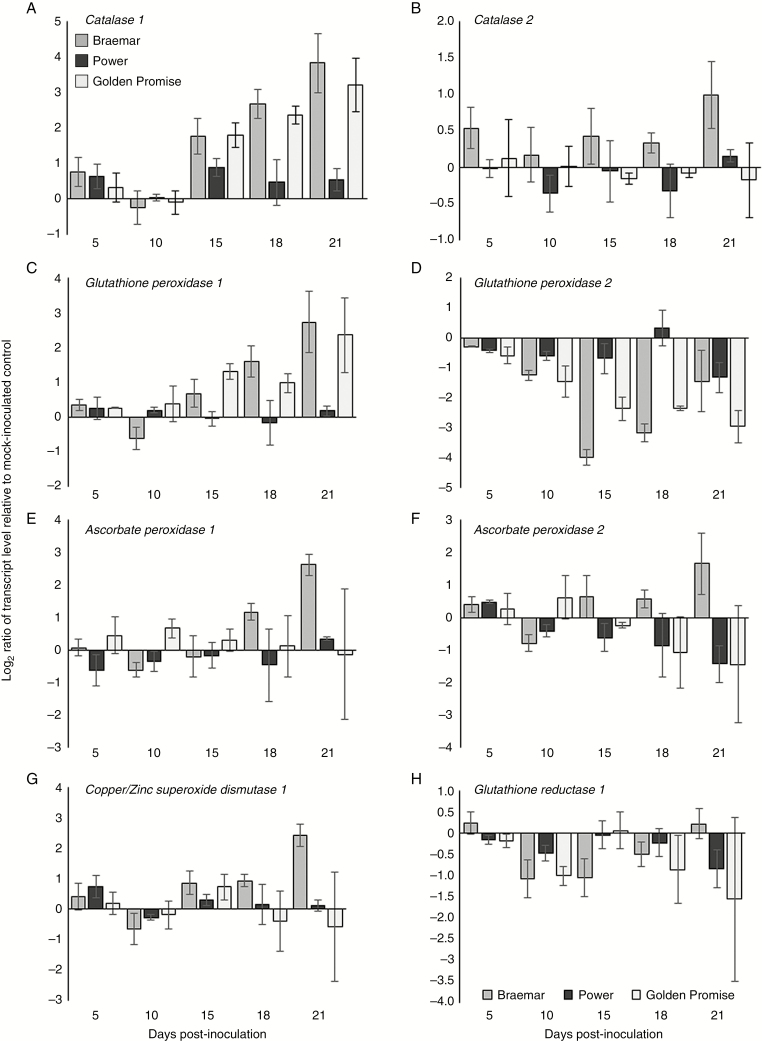
Expression analysis of host antioxidant transcript levels during the *Ramularia collo*-*cygni* infection time course. Transcript accumulation of (A) *Catalase 1*, (B) *Catalase 2*, (C) *Glutathione peroxidase 1*, (D) *Glutathione peroxidase 2*, (E) *Ascorbate peroxidase 1*, (F) *Ascorbate peroxidase 2*, (G) *Copper/zinc superoxide dismutase 1* and (H) *Glutathione reductase 1* in Braemar, Power and Golden Promise. Log_2_-transformed data are presented. Error bars indicate ±1 s.e.

The antioxidant system acts to protect the plant from oxidative damage caused by high levels of toxic ROS (Foyer and Noctor, 2005). No change in expression of any gene involved in the antioxidant system was observed in the partially resistant variety Power (Fig. 6). *Ramularia collo*-*cygni* infection reduced *Glutathione peroxidase 2* (*GPX2*) transcript accumulation from 10 dpi onwards in the susceptible varieties Braemar and Golden Promise (Fig. 6D). None of the other antioxidant genes was differentially expressed during asymptomatic development (5–10 dpi) in any of the cultivars tested (Fig. 6). There was increased expression of the major H_2_O_2_ and superoxide scavenger genes *Catalase 1* (*Cat1*) from 15 dpi and *Glutathione peroxidase 1* (*GPX1*) from 21 dpi in both Braemar and Golden Promise (Fig. 6A). Expression of *CSD1* was also elevated in Braemar at 21 dpi (Fig. 6G).

Transcript levels of *Pathogenesis-related 1* (*PR1*) increased in all three varieties at 10 dpi and remained high in both the susceptible varieties, especially Braemar, but not in Power (Supplementary Data Fig. S5A). Expression of the cell death suppressor *Bax-inhibitor 1* (BI-1) increased over time in the susceptible varieties Braemar and Golden Promise, but not significantly in Power (Supplementary Data Fig. S5B). Induction of *Mitogen-activated protein kinase 3* (*MPK3*) has been linked to host-induced cell death involved in the susceptibility of wheat to the related pathogen *Zymoseptoria tritici* (Rudd *et al.*, 2008). The MPK3 transcript was elevated at the later stage of infection in the highly susceptible variety Braemar but was not differentially expressed in either Power or Golden Promise (Supplementary Data Fig. S5C). There was no change in the expression of *Mitogen-activated protein kinase 6* (*MPK6*) in any of the varieties (Supplementary Data Fig. S5D).

### Effect of manipulation of the host antioxidant system on the development of Ramularia leaf spot

**Fig. 7.**
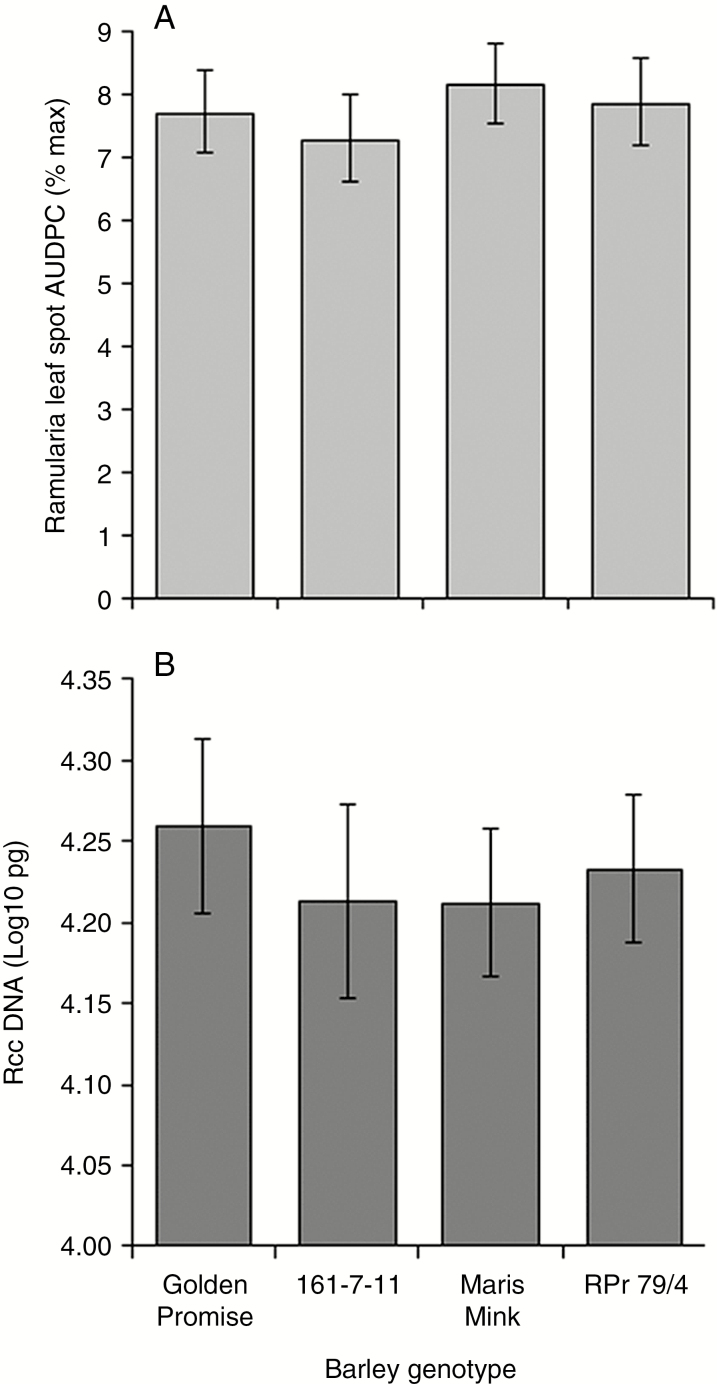
Development of Ramularia leaf spot on spring barley lines with impaired antioxidant systems. (A) Disease development on transgenic Golden Promise RNAi line 161-7-11 with reduced *Copper/zinc superoxide dismutase 1* transcript levels and the Maris Mink mutant RPr 79/4 with reduced catalase activity. Disease is expressed as the area under the disease progress curve (AUDPC). (B) *Ramularia collo*-*cygni* DNA levels in prophyll leaves 21 d post-inoculation measured by qPCR. Error bars indicate ±1 s.d. Data were analysed by general linear modelling. ***P < 0.001; **0.001 < *P* < 0.01; *0.01 < *P* < 0.05.

The effect of reduced host antioxidant capacity on the development of RLS was investigated using barley lines deficient in major ROS-scavenging enzymes. The transgenic RNAi line, 161-7-11, has an approx. 4-fold reduction in transcript levels of *CSD1* (Lightfoot *et al.*, 2017), whereas the mutant RPr 79/4 has an approx. 90 % reduction in leaf catalase activity (Kendall *et al.*, 1983). No significant differences in RLS symptom development were observed in either 161-7-11 or RPr 79/4 compared with their respective wild-type lines (Fig. 7A). Similar levels of *R*. *collo*-*cygni* DNA were recorded in both line 161-7-11 and RPr 79/4 compared with their respective wild-type lines at 21 dpi (Fig. 7B).

**Fig. 8.**
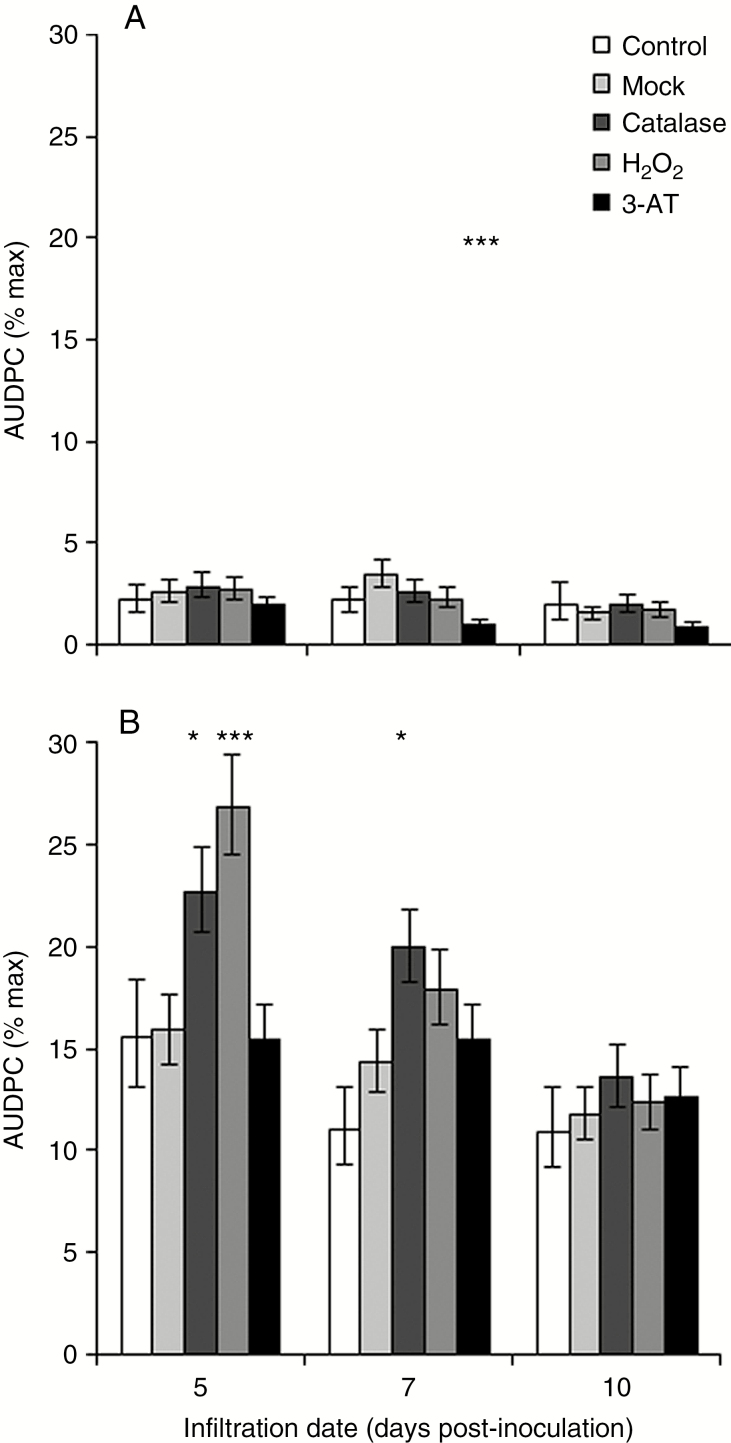
Effect of manipulation of H_2_O_2_ in barley leaves infected with *Ramularia collo*-*cygni*. Prophyll leaves of Power (A) and Braemar (B) were infiltrated with water (Control), 2000 U mL^–1^ catalase, 5 mm H_2_O_2_ or 5 mm 3-amino-1,2,4-triazole (3-AT), or uninfiltrated (control) at 5, 7 and 10 d post-inoculation with *R*. *collo*-*cygni* and the effect on disease development assessed by calculating the area under the disease progress curve (AUDPC). Error bars indicate ±1 s.e. Data were analysed using general linear modelling. ***P < 0.001; **0.001 < *P* < 0.01; *0.01 < *P* < 0.05 for comparisons with mock-inoculated leaves.

Next the effect of manipulating foliar H_2_O_2_ on RLS symptom development was tested. Neither catalase nor H_2_O_2_ infiltration affected disease development in Power significantly. Although the catalase inhibitor 3-AT significantly reduced RLS in Power at 7 dpi (Fig. 8A), 3-AT has a fungistatic effect *in vitro* (*P* < 0.001; Supplementary Data Fig. S6). Infiltration of catalase into the leaf apoplast at 5 or 7 dpi significantly (*P* < 0.05) increased disease severity in Braemar (Fig. 8B), as did infiltration of H_2_O_2_ at 5 dpi (*P* < 0.001), but no significant effect was seen 10 dpi. The partially RLS-resistant varieties Chevallier and Proctor also showed no significant RLS response to catalase or H_2_O_2_ infiltration, but neither did the moderately susceptible variety Optic (Supplementary Data Fig. S7). Catalase infiltration 5 dpi significantly increased RLS susceptibility in Ingrid (*P* < 0.05) and in IngridBC*mlo5* (Supplementary Data Fig. S7; *P* < 0.01). Golden Promise showed significantly increased RLS following infiltration with catalase (*P* < 0.01) or H_2_O_2_ (*P* < 0.05) at 5 dpi (Supplementary Data Fig. S7). However, the infiltration process itself appeared to affect disease development in this variety as infiltrated control leaves showed higher levels of disease than water-infiltrated mock-inoculated leaves (Supplementary Data Fig. S7; *P* < 0.01). Disease levels were also lower in Golden Promise leaves infiltrated with any treatment at 7 dpi. Infiltration with 3-AT reduced disease severity at one time point or more in all the varieties tested except Braemar (Fig. 8; Supplementary Data Fig. S7).

Changes to the host antioxidant systems appeared to affect disease development in RLS-susceptible varieties (Fig. 8; Supplementary Data Fig. S7). As *R*. *collo-cygni* produces toxins that induce ROS and tissue death (Heiser *et al.*, 2003), the sensitivity of barley varieties to ROS-induced lesion development was tested. The size of lesions formed by alloxan (*P* < 0.001), menadione (*P* < 0.001) and methyl viologen (*P* < 0.001) varied between barley varieties (Supplementary Data Fig. S8) but was not correlated with varietal susceptibility to RLS [correlation (*r*) between alloxan lesion size and mean logit-transformed RLS scores = 0.11, *P =* 0.8; menadione *r* = 0.67, *P* = 0.07; methyl viologen *r* = 0.34, *P* = 0.4].

### The effect of the *bst1* lesion mimic mutants on Ramularia leaf spot development

**Fig. 9.**
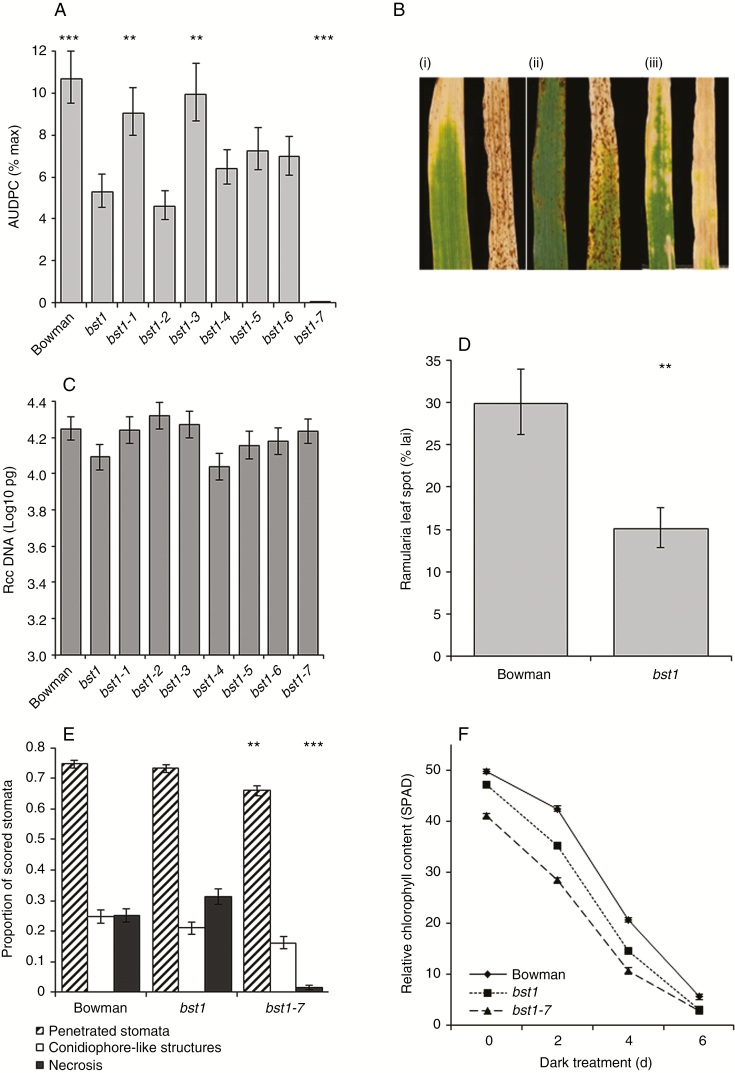
Effect of *Bipolaris sorokiniana tolerant 1* (*bst1*) mutations on Ramularia leaf spot development. (A) Disease development on *bst1* mutants. (B) Representative images of uninoculated (left) and *Ramularia collo*-*cygni*-inoculated wild-type Bowman (i), *bst1* (ii) and *bst1-7* (iii) leaves 21 d post-inoculation (dpi). Disease is expressed as the area under the disease progress curve (AUDPC). (C) *R*. *collo*-*cygni* DNA levels in prophyll leaves 21 dpi measured by qPCR. (D) Final levels of Ramularia leaf spot symptoms expressed as percent leaf area infected (% lai) on Bowman and *bst1* mutants 21 dpi. (E) Microscopic comparison of *R*. *collo*-*cygni* infection between Bowman, *bst1* and *bst1-7* at 21 dpi. (F) Dark-induced senescence assays for Bowman, *bst1* and *bst1-7*. Error bars indicate ±1 s.e. Pathology and qPCR data were analysed by general linear modelling. Dark-induced senescence data were analysed using a linear mixed model of repeated measures. ***P < 0.001; **0.001 < *P* < 0.01; *0.01 < *P* < 0.05 for comparisons with *bst1* (A and E) or Bowman (D).

The role of redox status and senescence in the development of RLS was further evaluated using the *bst1* lesion mimic mutant which has lower H_2_O_2_ levels during pathogen infection (Persson *et al.*, 2008, 2009). When inoculated with *R*. *collo*-*cygni*, the *bst1* mutant showed significantly reduced development of RLS symptoms compared with wild-type Bowman (Fig. 9A, B; *P* < 0.001) but not a significantly lower amount of *R*. *collo*-*cygni* DNA 21 dpi (Fig. 9C, *P* = 0.2). Despite the two lines having similar fungal DNA levels 21 dpi, *bst1* had fewer disease symptoms than Bowman (Fig. 9B, D; *P* = 0.002).

How the *bst1* mutation influences RLS development was further examined using a set of *bst1* double mutants that had undergone a second round of mutagenesis resulting in variation in the size, shape and number of spontaneous necrotic spots produced by each lesion mimic (Persson *et al.*, 2008). Some *bst1* double mutation lines displayed a differential response to RLS compared with the *bst1* mutant. The lines *bst1-2*, *bst1-4*, *bst1-5* and *bst1-6* all had disease levels comparable with *bst1* (Fig. 9A). *bst1-1* and *bst1-3* both showed significantly more RLS symptoms than *bst1*, similar to those on the wild-type line Bowman (Fig. 9A). No RLS symptoms were observed on the line *bst1-7*, but rather inoculated leaves of this line turned yellow and appeared to senesce completely in the absence of fungal symptoms (Fig. 9B). No significant difference in *R*. *collo*-*cygni* DNA levels was observed between the *bst1* mutant or any of the *bst1* double mutation lines, including *bst1-7* which did not exhibit any RLS symptoms (Fig. 9C; *P* = 0.2). Microscopic analysis of *R*. *collo*-*cygni* development 21 dpi indicated no significant differences in fungal penetration between the *bst1* mutant and Bowman (*P* = 0.5), but stomatal penetration was lower in the double mutant (*P* = 0.002). There was no significant difference in the production of conidiophore-like structures between *bst1* and either Bowman (*P* = 0.2) or *bst1-7* (*P* = 0.1; Fig. 9E). *bst1-7* mutants had much less cellular necrosis than *bst1* plants (Fig. 9E; *P* < 0.001). More necrosis was scored on the *bst1* mutants than on Bowman, but this was not statistically significant (Fig. 9E; *P* = 0.09). These results suggest that *R*. *collo*-*cygni* biomass accumulates normally on *bst1-7* plants, but the triggers required for changes in fungal development and to enter the necrotrophic, pathogenic phase are altered.

Both the *bst1* and *bst1-7* mutants exhibited a ≥2-fold reduction in expression of the antioxidant genes *APX1*, *APX2*, *GPX2* and *GR1*, and a >2-fold increase in expression of *Cat2* (Supplementary Data Fig. S9A) relative to wild-type plants. *bst1* and *bst1-7* plants also showed a very large increase in expression of the defence-related gene *PR1* (Supplementary Data Fig. S9B) but only a small reduction in expression of the cell death-related gene *BI-1* (Supplementary Data Fig. S9C) compared with Bowman. Dark-induced senescence assays were used to examine differences in leaf senescence between the two *bst* mutants and Bowman. Both *bst1* (*P* < 0.01) and *bst1-7* (*P* < 0.001) leaves had significantly lower SPAD readings at day 0, suggesting that these lines have a reduced chlorophyll content relative to the wild type, a trend that continued throughout the senescence time course (Fig. 9F).

### The role of functional chloroplasts in expression of Ramularia leaf spot symptoms

**Fig. 10.**
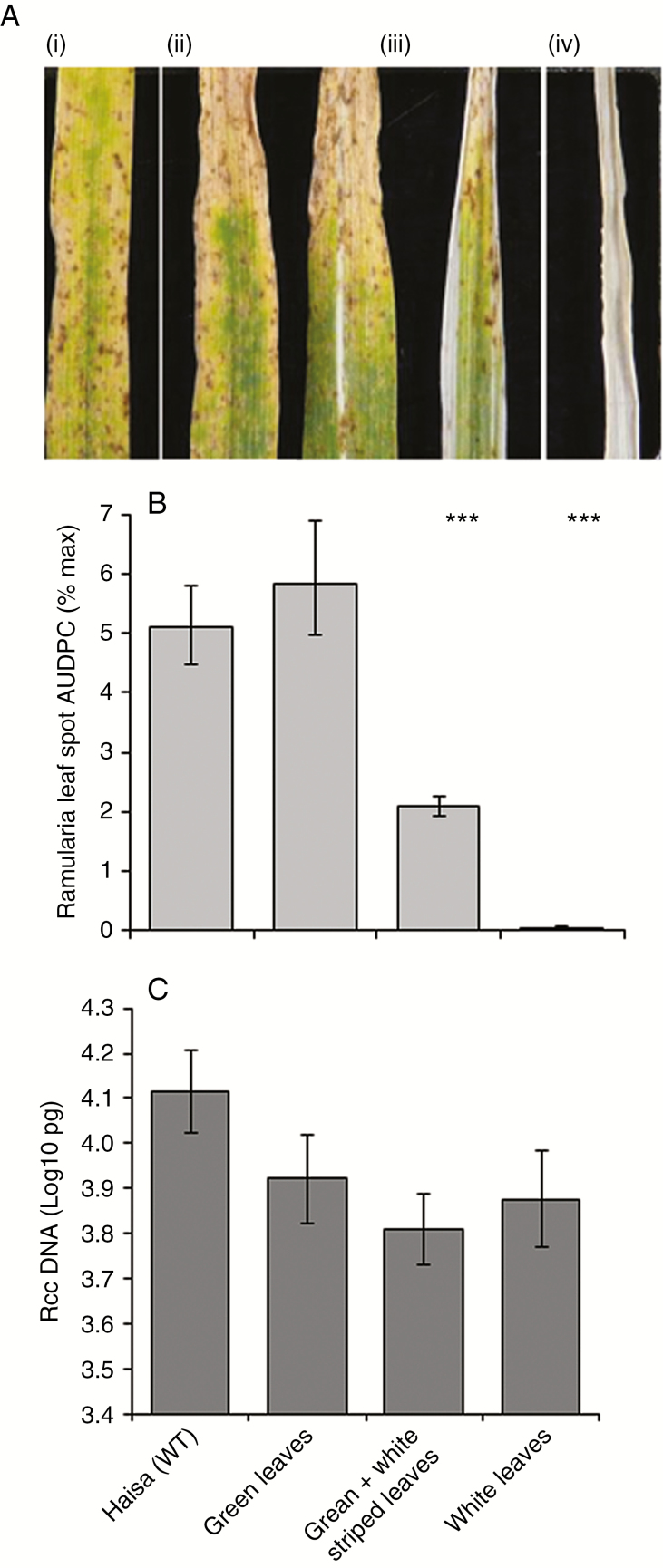
Development of Ramularia leaf spot on barley *albostrians* mutants. (A) Typical disease symptoms observed on the mother line Haisa (i), green-leafed *albostrians* mutants (ii), green and white-leafed *albostrians* mutants (iii) and white-leafed *albostrians* mutants (iv). (B) Disease development was measured as the area under disease progress curve (AUDPC). (C) *Ramularia collo*-*cygni* DNA levels in prophyll leaves 21 d post-inoculation measured by qPCR. Error bars indicate ±1 s.e. All data were analysed by general linear modelling. ***P < 0.001 for comparisons with green-leaved *albostrians* mutants.

The rapid onset of leaf chlorosis of *bst1-7*-inoculated leaves in the absence of RLS symptoms suggested that functional chloroplasts are required for *R*. *collo*-*cygni* to enter its pathogenic phase. This hypothesis was tested using the *albostrians* mutants which have defective chloroplast development (Hess *et al.*, 1994). Typical RLS lesions formed on wild-type Haisa [Fig. 10A (i)] and green *albostrians* [Fig. 10A (ii)] leaves and there was no significant difference in disease development between these lines (Fig. 10B). Classic RLS symptoms also formed on green and white striped leaves [Fig. 10A (iii)] but disease development was significantly lower on these leaves than on green leaves (Fig. 10B; *P* < 0.001). No RLS symptoms were observed on white leaved mutant plants [Fig. 10A (iv), 10B; *P* <0.001]. Despite the reduced development of disease symptoms in the green and white striped leaves and the complete absence of disease symptoms in plants with white leaves, there was no significant difference in the level of *R*. *collo*-*cygni* genomic DNA detected 21 dpi between any of the mutant lines (Fig. 10C; *P* = 0.1). Together these data suggest that plants with defective chloroplasts affect the ability of *R*. *collo*-*cygni* to cause disease.

## DISCUSSION

Ramularia leaf spot has become an important disease of barley in Europe and other temperate regions of the world in the last 20 years, but the fungus responsible for the disease, *R*. *collo*-*cygni*, has been associated with barley at least as far back as the 1800s (Fountaine and Fraaije, 2009; Havis *et al.*, 2015). Like its relatives within the Mycosphaerellaceae, *R*. *collo*-*cygni* has a prolonged latent growth phase on its host, with symptoms only observed late in the growing season, usually after flowering; in some years, disease symptoms fail to develop at all. This, together with horizontal transmission of the fungus through seed (Havis *et al.*, 2014) and other features of fungal development (Kaczmarek *et al.*, 2017), has led to the proposal that *R*. *collo*-*cygni* is an endophyte that only causes disease under specific circumstances (McGrann and Havis, 2017). This prompts the question of what factors cause *R. collo-cygni* to become a destructive pathogen. While RLS is typically most severe in the top two leaves of naturally infected, adult barley plants in field conditions (Schützendübel *et al.*, 2008), artificial inoculation of seedlings with *Rcc* was used in the experiments reported here as a tractable model system for investigating factors that induce symptom development.

Endophytes develop a balanced interaction with their host, but can become pathogenic when that balance is disturbed (Saikonnen, 2005; Schulz and Boyle, 2005). Almost all barley varieties tested showed increased RLS symptoms if plants had been subjected to abiotic stress before fungal infection, whether caused by high light or by waterlogging. The exceptions concerned two of the most resistant varieties, as Athena plants had lower symptoms following either form of abiotic stress while Blenheim plants exposed to high light prior to inoculation showed no change in RLS. This indicates that stress to the host alters the relationship with the *R*. *collo*-*cygni* fungus and elicits more extensive development of RLS symptoms, especially in susceptible genotypes. Increased RLS expression has been associated with stress caused by high light intensity (Makepeace *et al.*, 2008; Peraldi *et al.*, 2014) in seedlings and the developmental shift to flowering (Schützendübel *et al.*, 2008) in field-grown adult plants, both of which result in accelerated senescence (Supplementary Data Fig. S2) and altered ROS balance within the host (Zimmermann and Zentgraf, 2005; Jajic *et al.*, 2015). Light intensity affects the transition to disease of many fungal pathogens in the Mycosphaerellaceae, including symptom expression by *Z. tritici*, *Pseudocercospora fijienesis* and *Dothistroma septosporum* (Bradshaw, 2004; Keon *et al.*, 2007; Churchill, 2011).

*In vitro*, *R*. *collo*-*cygni* is tolerant of conditions that induce oxidative stress (Fig. 5) such as high H_2_O_2_ levels, which are associated with the later stages of disease (Figs 2 and 4; Supplementary Data Fig. S3). The role of H_2_O_2_ in RLS may, again, be similar to that in other diseases caused by fungi in the Mycosphaerellaceae. The development of Septoria tritici blotch symptoms in wheat caused by the fungus *Z. tritici*, which is closely related to *R. collo-cygni* (McGrann *et al.*, 2016), was defined by an accumulation of H_2_O_2_, PR proteins and fungal biomass (Yang *et al.*, 2013), similar to that observed in RLS (Figs 1C, 2 and 4; Supplementary Data Figs S3 and S5). *Ramularia collo*-*cygni* has an abundance of chloroperoxidase proteins which use H_2_O_2_ as a substrate, a common trait within the Mycosphaerellaceae (do Amaral *et al.*, 2012). Many chloroperoxidase genes are upregulated during asymptomatic development of *Z*. *tritici* (Rudd *et al.*, 2015), suggesting that they may be important in allowing the pathogen to develop in environments high in H_2_O_2_ such as plants under oxidative stress. It is feasible that *R*. *collo*-*cygni* uses a similar system to cope with high levels of this ROS during disease development.

It is not known why NBT staining was lower in Rcc09B4-inoculated leaves than in mock-inoculated leaves. At earlier time points (5–10 dpi), reduced NBT in inoculated material may indicate that the fungus acts to delay ROS production in the host during endophytic growth, whereas at later times it may reflect conversion of superoxide to H_2_O_2_, noting the strong DAB staining in susceptible varieties at these times (Fig. 4; Supplementary Data Fig. S3).

Expression of RLS symptoms appears to be linked to ROS status within the leaf. Mutations in genes such as *mlo*, *ror1*, *ror2*, *nec1* and *bst1* (Hückelhoven *et al.*, 2000; Piffanelli *et al.*, 2002; Persson *et al.*, 2009; McGrann *et al.*, 2014; McGrann *et al.*, 2015*b*) that result in mis-regulation of ROS all affect the transition from endophyte to necrotroph in barley seedlings. It is likely, however, that accumulation of H_2_O_2_ either is not responsible for the transition to RLS disease or acts together with other factors to elicit symptoms. On the one hand, the *bst1* mutant showed lower RLS levels than the wild type (Fig. 9) and accumulated reduced levels of H_2_O_2_ in response to pathogen challenge (Persson *et al.*, 2009). On the other hand, barley *nec1* mutants, which also show reduced RLS symptoms (McGrann *et al.*, 2015*b*), increased H_2_O_2_ levels in response to pathogen inoculation (Keisa *et al.*, 2011). Furthermore, no effect on RLS symptoms was observed in the catalase-deficient mutant RPr 79/4 (Kendall *et al.*, 1983) or the *HvCSD1* RNAi line (Lightfoot *et al.*, 2017; Fig. 7), both of which have reduced antioxidant levels potentially affecting foliar H_2_O_2_ levels.

Infiltration of *R*. *collo*-*cygni*-inoculated barley leaves with catalase or H_2_O_2_ prior to symptom formation increased symptom expression in most of the susceptible varieties tested, but not the partially resistant varieties (Fig. 8; Supplementary Data Fig. S7). This trend was not as consistent across varieties as the effect of abiotic stress treatments on disease expression (Fig. 3), possibly indicating that varieties vary in their ability to cope with a change in H_2_O_2_ status and thus in the development of RLS. In Septoria tritici blotch, in contrast to RLS, infiltration of wheat leaves with catalase increased susceptibility to *Z*. *tritici* whereas H_2_O_2_ infiltration increased resistance, demonstrating that manipulating the redox status in cereal leaves can alter the host response to this pathogen (Shetty *et al.*, 2007), although not necessarily in the same direction as *R. collo-cygni*. Note that *Z*. *tritici* was more sensitive to lower concentrations of H_2_O_2_ in the assays of Shetty *et al.* (2007) than *R*. *collo*-*cygni* (Fig. 5), which may explain why *R*. *collo*-*cygni* can remain asymptomatic throughout its life cycle in the field despite experiencing oxidative stress.

These data on the interaction of *R. collo-cygni* with the plant redox system suggest that expression of disease symptoms in seedlings is not associated with elevated ROS levels as such, but rather with changes in ROS status. This is consistent with the concept that in the endophytic stage of its life cycle, *R. collo-cygni* has a finely balanced interaction with its host plant, but a change in that balance in either direction may cause it to become a necrotrophic parasite. RLS development is associated with an increase in either H_2_O_2_ accumulation or catalase gene transcription, which is expected to accelerate reduction of H_2_O_2_, since infiltration with either of these reagents promotes symptom expression in more susceptible barley varieties. As such, disease caused by *R*. *collo*-*cygni* may reflect miscommunication between the pathogen and its host caused by changes in barley ROS levels rather than aggressive pathogenicity, as has been suggested for other endophytes (Rodriguez and Redman, 2008).

In the field, symptoms of RLS are typically observed in the later part of the growing season after the crop has flowered. Symptom expression in seedlings and adult plants has previously been linked with leaf senescence (McGrann *et al.*, 2015*a*), a general decline in leaf antioxidant status (Schützendübel *et al.*, 2008) and a concomitant increase in ROS (Fig. 4; Supplementary Data Fig. S3). Abiotic stress, such as high light, accelerates the onset of senescence in seedlings (Szechyńska-Hebda and Karpiński, 2013), but the data shown here indicate that there is not a simple correlation between the rate of senescence and susceptibility to RLS, implying that the rate of varietal senescence is not sufficient to account for variation in disease severity (Supplementary Data Fig. S2).

Gradual breakdown of the chloroplast and the resulting changes in ROS levels may therefore have key roles as signals to trigger the transition of *R*. *collo*-*cygni* to pathogenic development. In support of this hypothesis, when senescence was promoted but chloroplast degradation occurred slowly following abiotic stress treatments (Supplementary Data Fig. S2), disease symptom expression was enhanced in all but the most RLS-resistant barley varieties (Fig. 3). Conversely, when chloroplast degradation occurred rapidly, as in the *bst1-7* mutant (Fig. 9), or did not occur, as in the *albostrians* mutants that have undifferentiated plastids (Fig. 10), *R*. *collo*-*cygni* remained in an endophytic stage and did not cause disease. This mechanism may be important in pathogenesis of other Mycosphaerellaceae fungi. Wheat plants in which defective chloroplast function was produced by silencing the carotenoid or chlorophyll biosynthesis genes *Phytoene desaturase* and *Magnesium chelatase H subunit* had reduced symptom production and less asexual reproduction by *Z*. *tritici* (Lee *et al.*, 2015). They also showed accelerated pathogen-induced ROS production, further suggesting an important role for chloroplast function and ROS in the transition of Mycosphaerellaceae fungi to necrotrophic parasitism.

The data presented here suggest that abiotic stress, senescence and elevated levels of ROS are not the proximate causes of the transition of *R. collo-cygni* from endophyte to necrotroph in artificially inoculated barley seedlings. Rather, ROS-mediated processes that lead to senescence may initiate the pathogenic transition of *R*. *collo*-*cygni* and thus RLS symptom development. Further insights into mechanisms that determine host susceptibility to RLS in seedlings will provide a better understanding of the factors that contribute to the aetiology of RLS in the field. This may lead to novel strategies for reducing losses of yield and grain quality caused by this aggressive new disease, including methods of selecting RLS-resistant barley varieties.

## SUPPLEMENTARY DATA

Supplementary data are available at https://academic.oup.com/aob and consist of the following. Table S1: barley varieties used in pathology experiments. Table S2: qRT-PCR primers used in this study. Figure S1: expression of chlorophyll *a*/*b*-binding protein in spring barley varieties Braemar, Power and Golden Promise following *Ramularia collo*-*cygni* inoculation. Figure S2: dark-induced senescence of prophyll leaves of the spring barley varieties Power, Braemar, Golden Promise, Chevallier, Optic, Proctor, Ingrid and IngridBCmlo5 grown under standard controlled environment room conditions or under high light conditions. Figure S3: ROS accumulation in Golden Promise following *Ramularia collo*-*cygni* inoculation. Figure S4: effect of oxidative stress-inducing media on *in vitro* growth of *Magnaporthe oryzae*, *Fusarium culmorum*, *Oculimacula yallundae* and *Botrytis cinereal*. Figure S5: expression analysis of defence-related gene transcript levels during the *Ramularia collo*-*cygni* infection time course. Figure S6: effect of reagents used to manipulate leaf H_2_O_2_ status on *in vitro* growth of *Ramularia collo*-*cygni*. Figure S7: effect of manipulation of H_2_O_2_ in barley leaves infected with *Ramularia collo*-*cygni.* Figure S8: lesion development caused by the reactive oxygen species donors alloxan, menadione and methyl viologen in different spring barley varieties. Figure S9: expression analysis of antioxidant- and defence-related gene transcript levels in Bowman, *Bipolaris sorokiniana tolerant 1* (*bst1*) and *bst1-7*.

aob-17235-s02

aob-17235-s03

aob-17235-s04

aob-17235-s05

aob-17235-s06

aob-17235-s07

aob-17235-s08

aob-17235-s09

aob-17235-s10

aob-17235-s11

aob-17235-s12

Supplemental-Data
