# Supplementary material for: The role of reactive oxygen in the development of Ramularia leaf spot disease in barley seedlings
Source: Ann Bot. 2017 Dec 22;121(3):415–30. doi: 10.1093/aob/mcx170 (PMC5838821; doi:10.1093/aob/mcx170)
Supplement: aob-17235-s02 [file mcx170_suppl_aob-17235-s02.docx]

Table S1 Barley varieties used in pathology experiments

| Variety | Response to Ramularia leaf spot |
| --- | --- |
| Braemar | Highly susceptible |
| Power | Partially resistant |
| Golden Promise | Moderately susceptible |
|  |  |
| Optic | Moderately susceptible |
| Chevalier | Partially resistant |
| Proctor | Partially resistant |
| Ingrid | Moderately susceptible |
| IngridBC*mlo5* | Highly susceptible |
|  |  |
| Athena | Partially resistant |
| Blenhiem | Partially resistant |
| Cooper | Susceptible |
| Kirsty | Moderately susceptible |
| Oxbridge | Partially resistant |
| Ria | Partially resistant |
| Tavern | Partially resistant |
| Pallas | Susceptible |
| P22 (PallasBC*mlo5*) | Highly susceptible |
|  |  |

Date on varietal responses to Ramularia leaf spot based on McGrann et al. (2014), Makepeace et al. (2008), Oxley et al. (2008) and unpublished observations.
