# Supplementary material for: The role of reactive oxygen in the development of Ramularia leaf spot disease in barley seedlings
Source: Ann Bot. 2017 Dec 22;121(3):415–30. doi: 10.1093/aob/mcx170 (PMC5838821; doi:10.1093/aob/mcx170)
Supplement: aob-17235-s03 [file mcx170_suppl_aob-17235-s03.docx]

Supplementary data Table S2 qRT-PCR primers used in this study

| Target gene | Accession | Target code | Forward primer | Reverse primer | Amplicon |
| --- | --- | --- | --- | --- | --- |
| Catalase 1 | U20777.1 ^1^ | *HvCAT1* | CCCGTCTGGAACAACAAC | CCCCGTGCATGAACAAC | 134 ^a^ |
| Catalase 2 | U20778.1 ^1^ | *HvCAT2* | CGACGACAAGATGCTGCAGT | TGGTTGTTCTTGAAGCCGC | 120 ^b^ |
| Glutathione peroxidase 1 | AJ238745.1 ^1^ | *HvGPX1* | AACGGCAACAATGTTTCTCC | ACAACGTGACCCTCCTTGTC | 119 ^a^ |
| Glutathione peroxidase 2 | AJ238744.1 ^1^ | *HvGPX2* | ACGTGAATGGCAACAATGCT | ATGACATGCCCCTCTTTGTC | 124 ^a^ |
| *Glutathione reductase 1* | AB277096.1 ^1^ | *HvGR1* | GGGGCTATAGTGGTCGATGA | AATGCTCCACCTTCCATCAG | 116 ^a^ |
| *Ascorbate peroxidase 1* | AJ006358 ^1^ | *HvAPX1* | CGGAGCTTTTGAGTGGTGACA | CCGCAGCATATTTCTCCACAA | 105 ^b^ |
| *Ascorbate peroxidase 2* | AK253050.1 ^1^ | *HvAPX2* | CGCCGAGAAGAACTGC | GCCGGTCTTGGTGGC | 82 ^a^ |
| *Copper-zinc superoxide dismutase 1* | KU179439.1 ^1^ | *HvCSD1* | ACCTCGGAAATGTGACAGC | ACCCTTGCCAAGATCATCAG | 140 ^a^ |
| *Pathogenesis-related protein 1* | TC203940 ^2^ | *HvPR1* | AGCACGAAGCTGCAGGCGTA | TCTCGTCCACCCACAGCTTCAC | 160 ^c^ |
| *Bax-inhibitor 1* | AJ290421.1 ^1^ | *HvBI-1* | GGCAGCTTCATGTTTGAGGT | AGGGCGTGCTTGATGTAGTC | 122 |
| *Mitogen-activated protein kinase 3* | TC199399 ^2^ | *HvMPK3* | TACCTAGAGCGGCTTCACGA | GTATCGGAAGTTGGGGTTCA | 147 |
| *Mitogen-activated protein kinase 6* | AK376245.1 ^1^ | *HvMPK6* | ATGGAGCTCATTGGAACACC | ATGATTGCCTTGCATGACG | 100 |
| Reference genes |  |  |  |  |  |
| Cyclophilin | CV056520 ^1^ | *HvCyclophilin* | TTGAGGACGAGATAAGGCCAG | GCGACTGACAAGGTGCAAGAG | 120 ^c^ |
| Elongation factor 1a | TC146566 ^2^ | *HvEF1a* | ATGATTCCCACCAAGCCCAT | ACACCAACAGCCACAGTTTGC | 101 ^c^ |
| Glyceraldehyde 3-phosphate dehydrogenase | M36650 ^1^ | *HvGAPDH* | CCTTCCGTGTTCCCACTGTTG | ATGCCCTTGAGGTTTCCCTC | 124 ^c^ |
| Tubulin A | AK252410.1 ^1^ | *HvTUBA* | AGTGTCCTGTCCACCCACTC | AGCATGAAGTGGATCCTTGG | 248 ^d^ |
| Ubiquitin | AK2050942.1 ^1^ | *HvUbiquitin* | GCCGCACCCTCGCCGACTAC | CGGCGTTGGGGCACTCCTTC | 219 ^e^ |

^1^ GenBank, ^2^ DFCI gene index

^a^ McGrann et al. (2015a), ^b^ Shagimardova et al. (2010), ^c^ McGrann et al. (2009), ^d^ Burton et al. (2004), ^e^ Rostoks et al. (2003)
