# Supplementary figures and images for: The role of reactive oxygen in the development of Ramularia leaf spot disease in barley seedlings
Source: Ann Bot. 2017 Dec 22;121(3):415–30. doi: 10.1093/aob/mcx170 (PMC5838821; doi:10.1093/aob/mcx170)

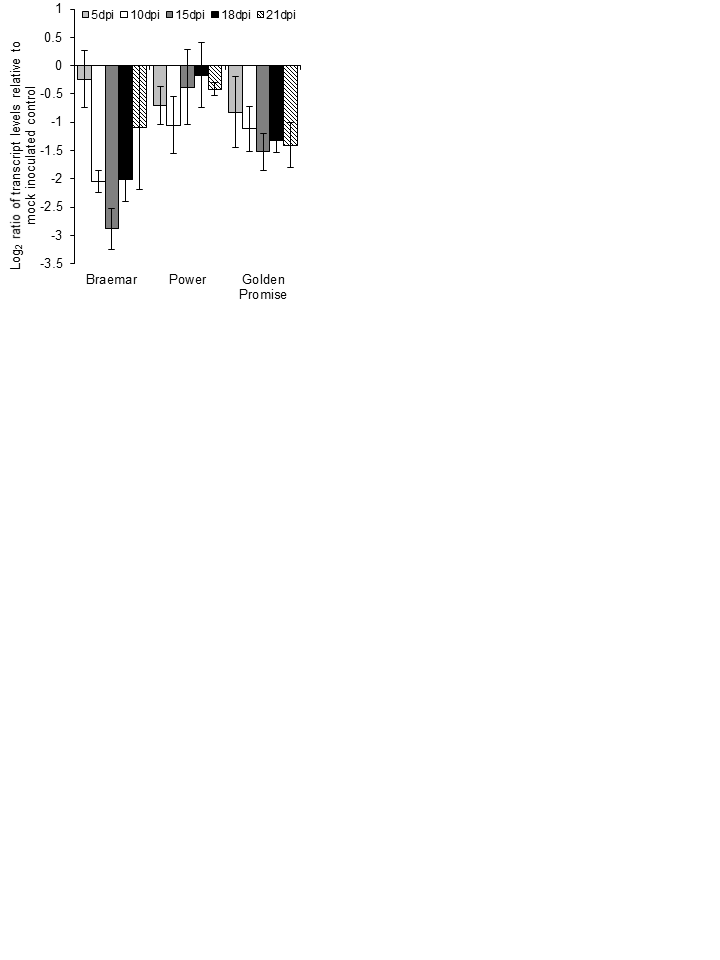

Supplement: aob-17235-s04 [file mcx170_suppl_aob-17235-s04.png]

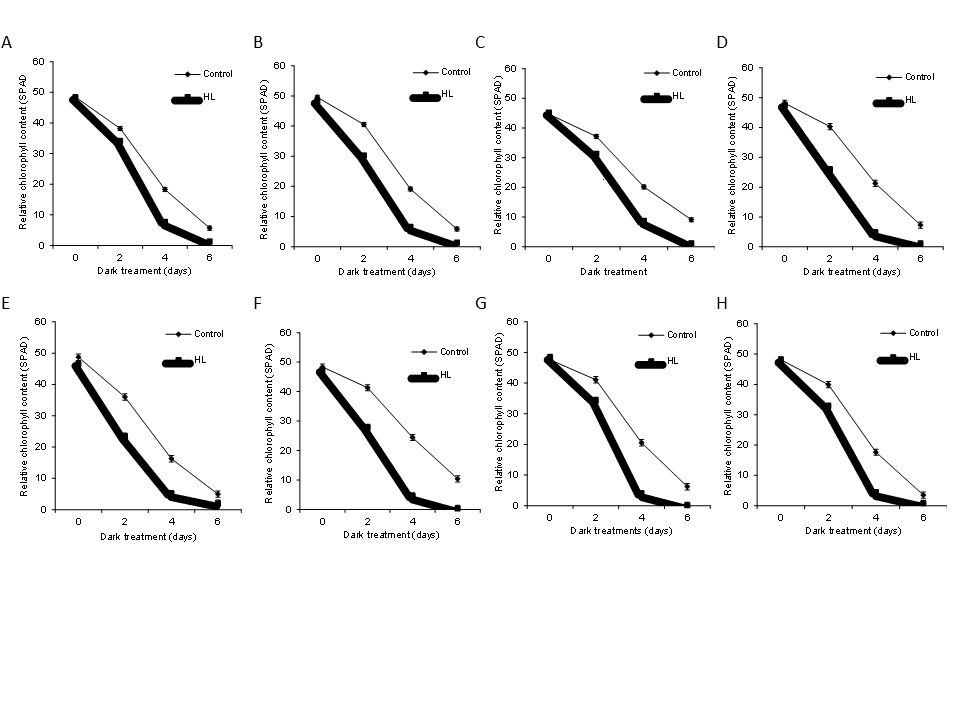

Supplement: aob-17235-s05 [file mcx170_suppl_aob-17235-s05.png]

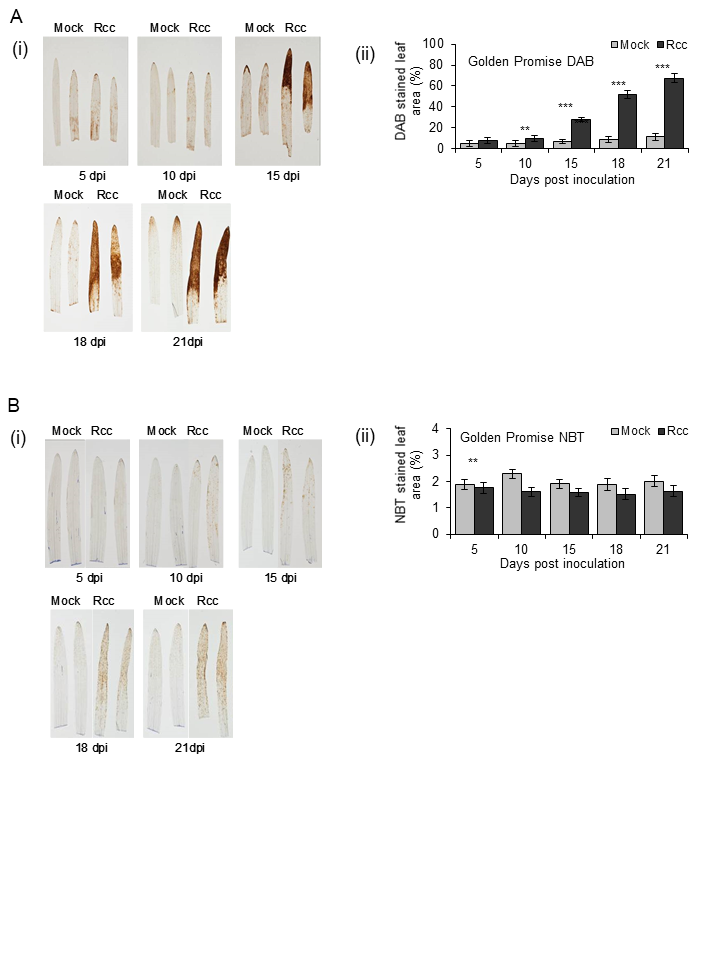

Supplement: aob-17235-s06 [file mcx170_suppl_aob-17235-s06.png]

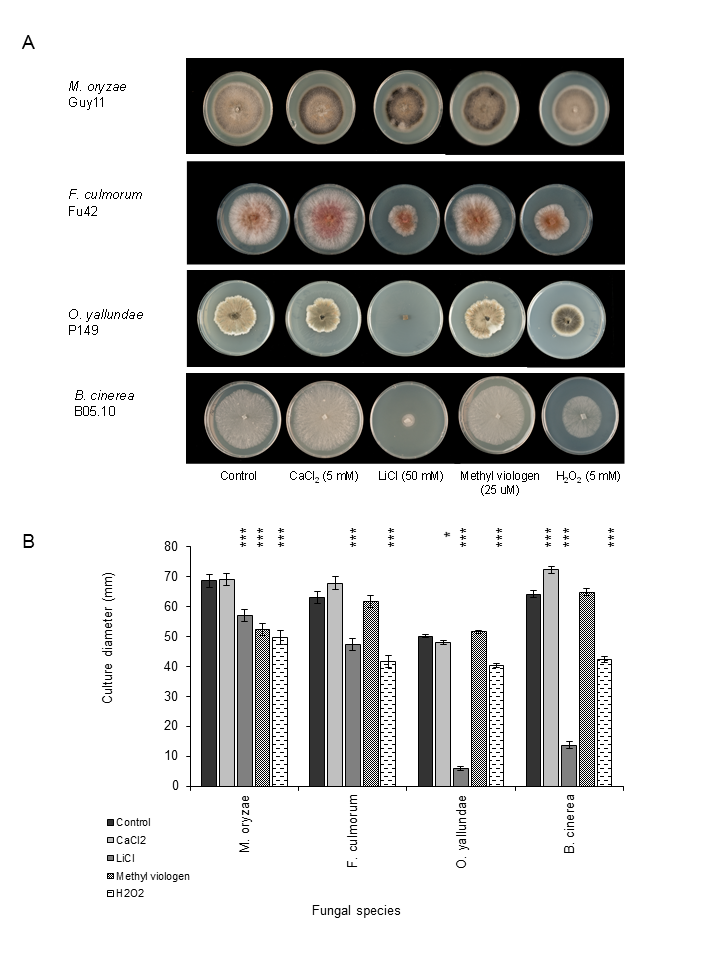

Supplement: aob-17235-s07 [file mcx170_suppl_aob-17235-s07.png]

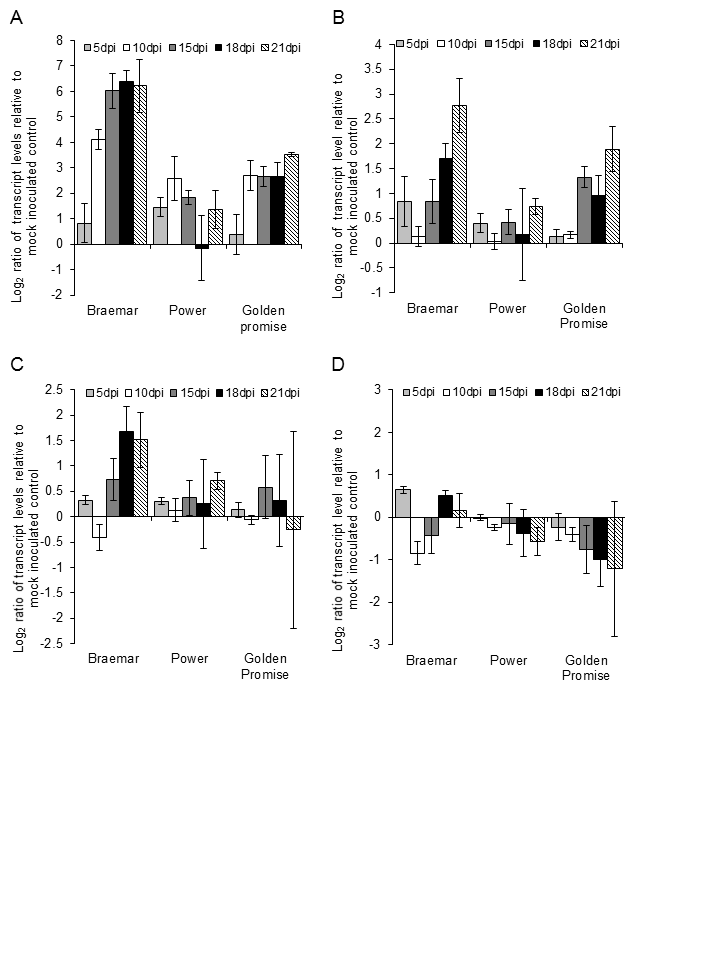

Supplement: aob-17235-s08 [file mcx170_suppl_aob-17235-s08.png]

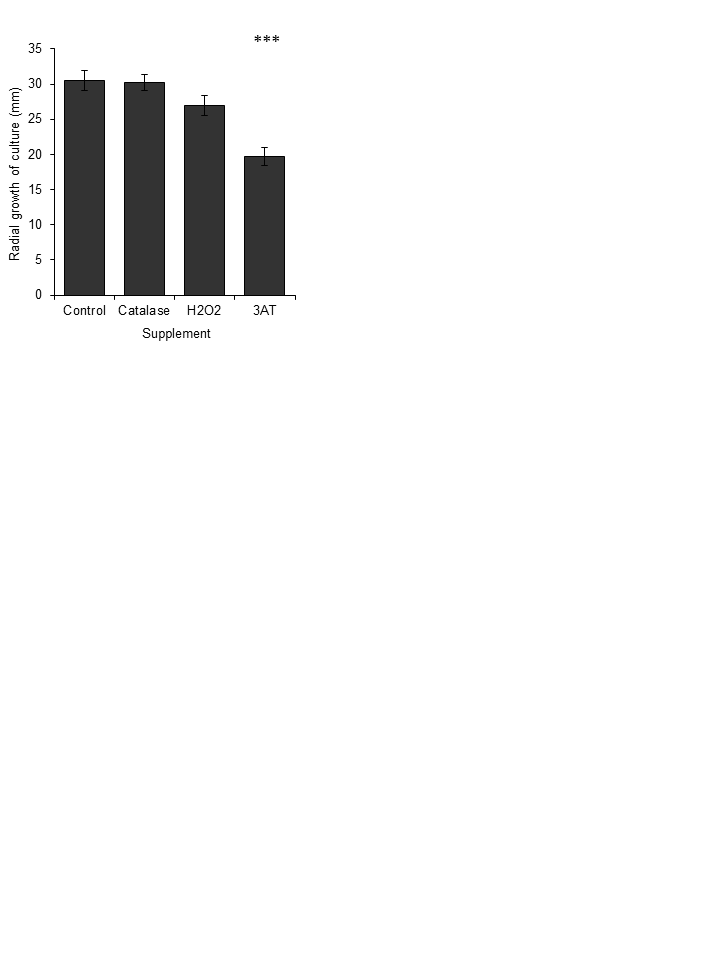

Supplement: aob-17235-s09 [file mcx170_suppl_aob-17235-s09.png]

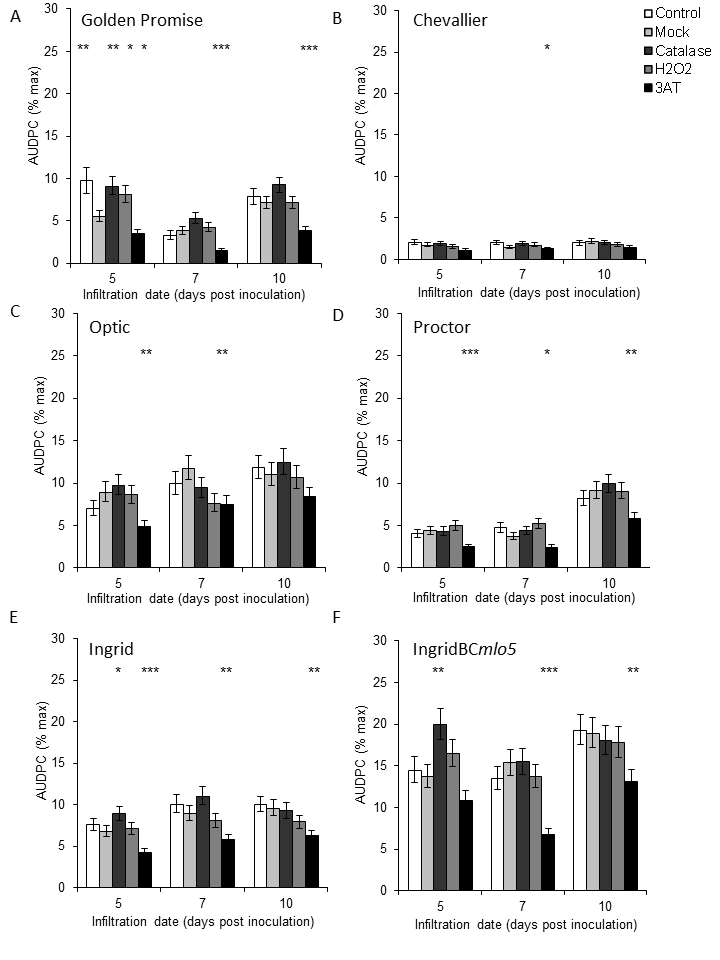

Supplement: aob-17235-s10 [file mcx170_suppl_aob-17235-s10.png]

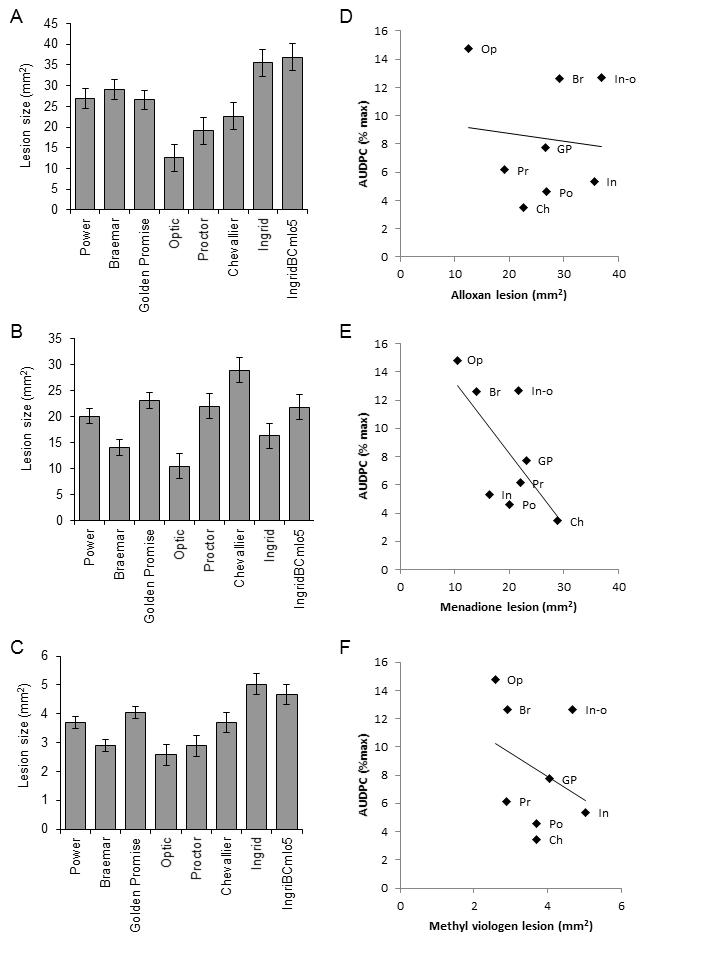

Supplement: aob-17235-s11 [file mcx170_suppl_aob-17235-s11.png]

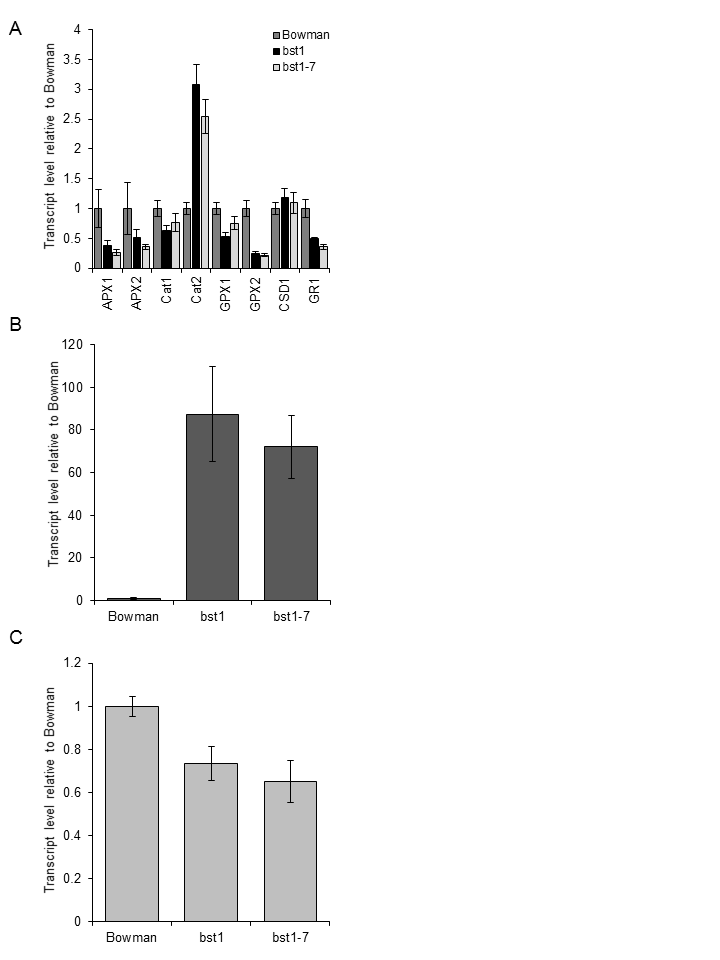

Supplement: aob-17235-s12 [file mcx170_suppl_aob-17235-s12.png]
