# Supplementary material for: The role of reactive oxygen in the development of Ramularia leaf spot disease in barley seedlings
Source: Ann Bot. 2017 Dec 22;121(3):415–30. doi: 10.1093/aob/mcx170 (PMC5838821; doi:10.1093/aob/mcx170)
Supplement: Supplemental-Data [file mcx170_suppl_supplemental-data.docx]

SUPPLEMENTAL DATA

The following supplemental materials are available:

Supplementary data Table S1 Barley varieties used in pathology experiments

Supplementary data Table S2 qRT-PCR primers used in this study

Supplementary data Figure S1 Expression of chlorophyll a/b binding protein in spring barley varieties Braemar, Power and Golden promise following *Ramularia* *collo*-*cygni* inoculation. Data shown relative to mock inoculated controls. Error bars indicate ±1 SE.

Supplementary data Figure S2 Dark-induced senescence of prophyll leaves of the spring barley varieties (A) Power (B) Braemar (C) Golden promise (D) Chevallier (E) Optic (F) Proctor (G) Ingrid (H) IngridBCmlo5 grown under standard controlled environment room conditions or under high light (HL) conditions. Error bars indicate ±1 SE.

Supplementary data Figure S3 Reactive oxygen species accumulation in Golden Promise following *Ramularia* *collo*-*cygni* inoculation. 3’3-diaminobenzidine (DAB) staining for peroxide accumulation (A) and Nitroblue tetrazolium (NBT) staining for superoxide accumulation (B). Representative images of DAB or NBT stained leaves (i) and proportion of leaves stained with DAB or NBT (ii) in mock and *R*. *collo*-*cygni* inoculated leaves. Error bars indicate ±1 SE. Data were analysed using a generalized linear model. ^***^ *P* <0.001; **0.001< *P* <0.01; * 0.01< *P* <0.05 for differences between inoculated and mock-inoculated samples.

Supplementary data Figure S4 Effect of oxidative stress-inducing media on *in vitro* growth of *Magnaporthe* *oryzae*, *Fusarium* *culmorum*, *Oculimacula* *yallundae* and *Botrytis* *cinerea*. Representative image (A) and measurements (B) of fungal culture diameter after 21 days growth of *M*. *oryzae* and *O*. *yallundae*, five days growth of *F*. *culmorum* and three days growth of *B*. *cinerea* on PDA media supplemented with CaCl_2_ (5 mM), LiCl (50 mM), H_2_O_2_ (5 mM) and methyl viologen (25 uM). Error bars indicate ±1 SE. Data were analysed by general linear modelling. ^***^ *P* <0.001; * 0.01< *P* <0.05 for differences from control plates with no supplement.

Supplementary data Figure S5 Expression analysis of defence-related gene transcript levels during *Ramularia* *collo*-*cygni* infection time course. Transcript accumulation of (A) *Pathogenesis-related protein 1* (PR1), (B) *Bax-inhibitor 1* (BI-1), (C) *Mitogen-activated protein kinase 3* (MPK3) and (D) *Mitogen-activated protein kinase 6* (MPK6) in Braemar, Power and Golden Promise. Log2 transformed data presented. Error bars indicate ±1 SE.

Supplementary data Figure S6 Effect of reagents used to manipulation leaf H_2_O_2_ status on *in vitro* growth of *Ramularia* *collo*-*cygni*. Measurements of *R*. *collo*-*cygni* culture diameter after 42 days growth on PDA media supplemented with catalase (2000 units mL^-1^), H_2_O_2_ (5 mM) and 3-amino-1,2,4-triazole (3AT; 5 mM). Error bars indicate ±1 SE. Data were analysed by general linear modelling. ^***^ *P* <0.001 for differences from control plates with no supplement.

Supplementary data Figure S7 Effect of manipulation of H_2_O_2_in barley leaves infected with *Ramularia* *collo*-*cygni*. Prophyll leaves of (A) Golden Promise (B) Chevallier (C) Optic (D) Proctor (E) Ingrid (F) IngridBC*mlo5* were infiltrated with water (mock-inoculation control), 2000 units mL^-1^ catalase, 5 mM H_2_O_2_or 5 mM 3-amino-1,2,4-triazole (3AT) or uninfiltrated (control) at 5, 7 and 10 days post inoculation with *R*. *collo*-*cygni* and the effect on disease development assessed by calculating the area under the disease progress curve (AUDPC). Error bars indicate ±1 SE. Data were analysed by general linear modelling. ^***^ *P* <0.001; ^**^ 0.001< *P* <0.01; * 0.01< *P* <0.05 for differences from mock-inoculated plants.

Supplementary data Figure S8 Lesion development caused by the reactive oxygen species donor’s alloxan (A), menadione (B) and methyl viologen (C) in different spring barley varieties. Comparison of varieties’ disease scores under standard controlled environment conditions and lesion size caused by alloxan (D), menadione (E) and methyl viologen (F).Codes are the first two letters of the varieties’ names; In-o, Pa-o: *mlo5* near-isogenic lines of Ingrid and Pallas respectively Error bars indicate ±1 SE.

Supplementary data Figure S9 Expression analysis of antioxidant and defence-related gene transcript levels in Bowman, *Bipolaris sorokiniana tolerant 1* (*bst1*) and *bst1-7*. Transcript accumulation of (A) antioxidant genes: *Ascorbate peroxidase 1* (APX1), *Ascorbate peroxidase 1* (APX2), *Catalase 1* (Cat1), *Catalase 2* (Cat2), *Glutathione peroxidase 1* (GPX1), *Glutathione peroxidase 2* (GPX2), *Copper-zinc superoxide dismutase 1* (CSD1) and *Glutathione reductase 1* (GR1), (B) *Pathogenesis-related protein 1*, (C) *Bax-inhibitor 1* (BI-1). Transcript levels shown relative to the wild type plant Bowman. Error bars indicate ±1 SE.
